# Protein chirality as a determinant of ligand affinity: insights from l- and d-streptavidin

**DOI:** 10.1039/d5sc06380a

**Published:** 2025-10-28

**Authors:** Riley J. Giesler, Peter C. S. Woodham, Steven R. E. Draper, Paul Spaltenstein, Frank G. Whitby, Christopher P. Hill, Michael S. Kay

**Affiliations:** a Department of Biochemistry, University of Utah 15 North Medical Drive East, Room 4100 Salt Lake City UT 84112 USA kay@biochem.utah.edu

## Abstract

Streptavidin enjoys numerous biotechnological applications due to its extraordinarily high affinity for biotin and the ease of biotinylation of targets through chemical or biological means. However, two main drawbacks limit its use in therapeutic and diagnostic applications: high immunogenicity and endogenous biotin interference. We propose that a mirror-image biotin/streptavidin system could solve these problems due to the minimal immunogenicity of mirror-image (d-) proteins and the expected lower binding affinity between non-natural d-streptavidin and natural d-biotin. To comprehensively address this problem, we first synthesized the l- and d-enantiomers of streptavidin using a three-segment native chemical ligation approach. This synthesis was enabled by temporarily solubilizing an aggregation-prone peptide segment with a Glu-based AlHx ‘helping hand’ linker. We developed a novel high-efficiency folding protocol and characterized the synthetic proteins by circular dichroism, size-exclusion chromatography, and binding to natural d-(+)-biotin and the mirror-image l-(−)-biotin *via* isothermal titration calorimetry. We found a 200-million-fold difference in affinity between streptavidin and its matched *vs.* mismatched biotin enantiomers that renders these systems functionally orthogonal. To gain further insight into how (−)-biotin binds recombinant streptavidin, we solved high-resolution X-ray crystal structures for both the matched and mismatched interactions. This work demonstrates the high degree of stereospecificity of the streptavidin/biotin interaction and the potential utility of a mirror-image biotin/streptavidin system for therapeutic and diagnostic applications.

## Abstract 2

Immunogenicity and endogenous biotin interference limit streptavidin's therapeutic/diagnostic potential. Synthesized l- and d-streptavidin demonstrate high stereospecificity, supporting the utility of a mirror-image biotin/streptavidin system.

## Graphical abstract

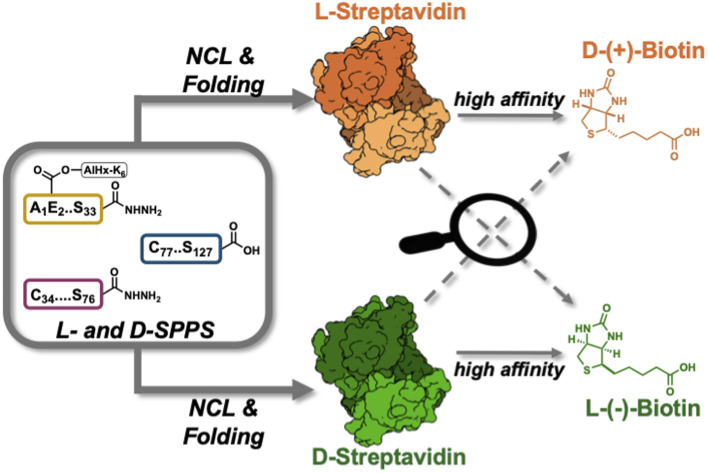

## Introduction

Streptavidin (SA), with its high solubility, ease of production in *E. coli*, and nearly irreversible affinity for biotin (40 fM)^1^ is an essential tool in biotechnology, including applications in immunoassays,^2^ affinity chromatography,^3^ phage display,^4^ proximity labeling,^5–7^ and diagnostic tests.^8^ SA has also been used for therapeutic applications,^9–15^ but none of these endeavors has progressed beyond early-stage clinical trials, likely due to the strong immunogenicity of SA as a bacterial protein and subsequent antibody-induced rapid clearance.^15–17^

**Fig. 1:**
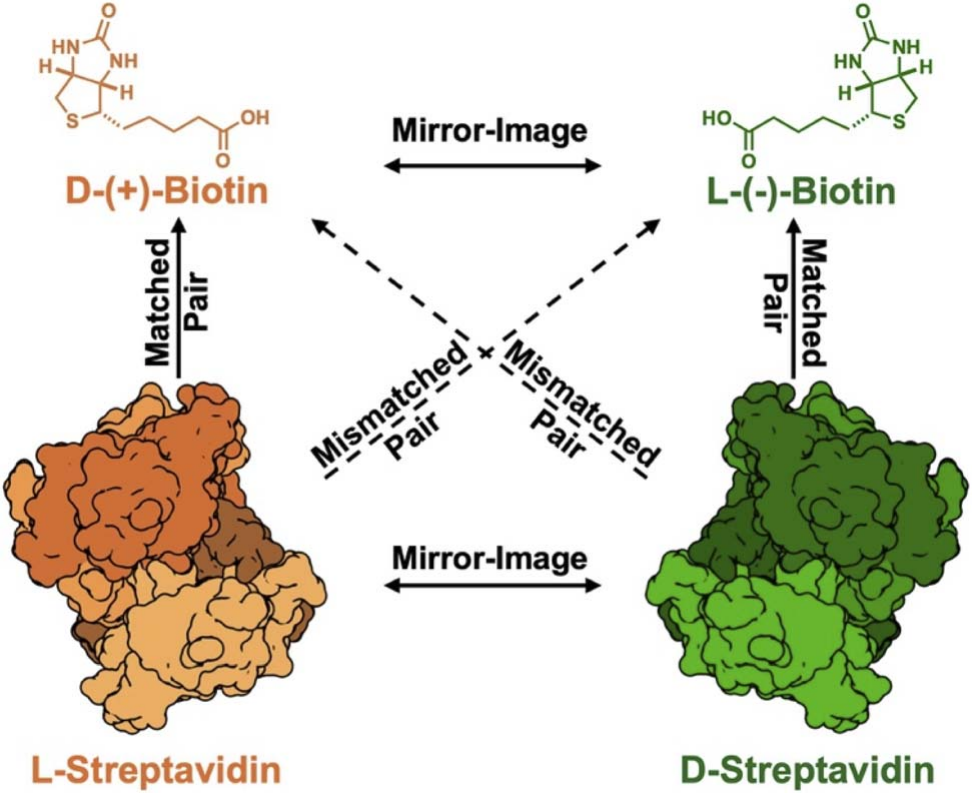
Summary of the different binding interactions described in this paper. The natural matched pair (orange) consists of the protein l-streptavidin and its ligand d-(+)-biotin. The mirror-image matched pair (green) is d-streptavidin with the ligand l-(−)-biotin. Streptavidin structures were adapted from PBD:1SWA.

A potential solution to this challenge would be the utilization of mirror-image SA (d-SA) and its corresponding non-natural ligand, l-(−)-biotin (Fig. 1). d-Proteins are minimally immunogenic since they cannot be proteolyzed by natural proteases for presentation by the major histocompatibility complex (MHC).^18,19^ Use of the mirror-image biotin-SA system could also overcome the interference of endogenous biotin (d-(+)-biotin) that can complicate the interpretation of results in applications such as diagnostic tests and proximity labeling.^2^ The law of mirror-image symmetry^20^ predicts that d-SA and (−)-biotin will share the same affinity as the natural matched binding pair (l-SA and (+)-biotin) (Fig. 1). This prediction does not provide insight into the potential cross-binding between the mismatched pairs, unnatural (−)-biotin to natural l-SA or natural (+)-biotin to unnatural d-SA. Suganama reported that (+)-biotin does not show binding to d-SA at concentrations up to 4 nM (surface plasmon resonance) and 100 nM (competition ELISA).^21^ However, individuals who supplement with biotin can have serum levels of ∼50–200 nM, which can lead to significant interference in both diagnostic and therapeutic applications.^22^ Furthermore, other synthesis-enabled studies exploring matched and mismatched protein-substrate pairs have shown that these interactions can have a degree of cross reactivity and could benefit from a more stringent investigation.^23^ For mirror SA/biotin technology to have maximum impact, this system must have negligible cross-binding between the mismatched pairs, which motivated us to systematically investigate this interaction and determine if any cross-binding occurs.

d-Proteins must be prepared through chemical protein synthesis (CPS), which can be accomplished by combining solid-phase peptide synthesis (SPPS)^24^ to make segments of ∼50 residues in length and a ligation chemistry, such as native chemical ligation (NCL), to access the full-length protein.^25^ NCL requires two functional groups: a C-terminal thioester on the acyl donor peptide and an N-terminal Cys on the acyl acceptor peptide. After the transthioesterification reaction between these groups, a rapid and irreversible S-to-N transfer occurs, resulting in native amide bond formation to join the two peptides. Due to the low abundance of Cys (1.4%),^26^ most CPS projects require introducing Cys at a native Ala site (8.2%)^26^ for NCL purposes followed by desulfurization to regenerate the native Ala.^27–31^

**Fig. 2:**
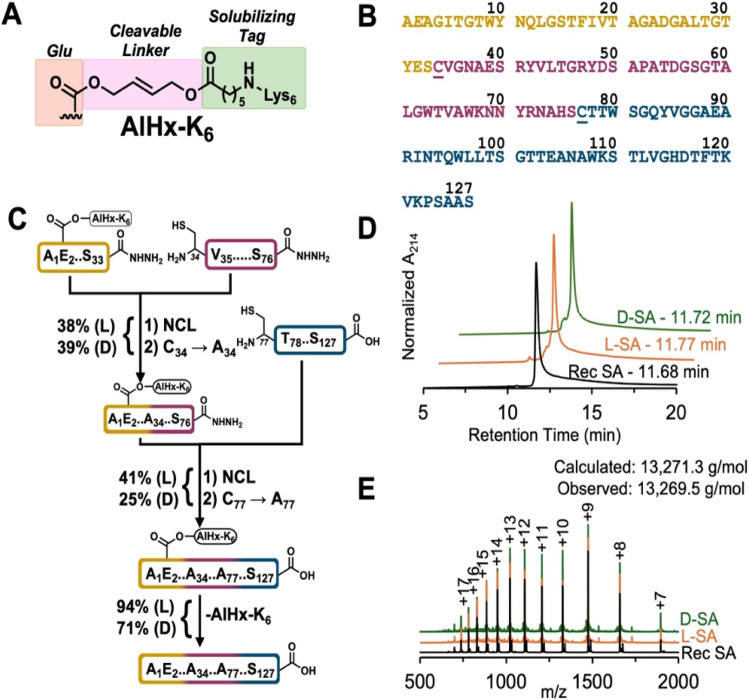
The AlHx-mediated assembly scheme of l- and d-streptavidin. (A) Structure of AlHx-K_6_ linker used in this synthesis. The allyl bridging group (pink) is stable to all CPS conditions and allows for Pd-mediated removal at the desired point. (B) Sequence of core streptavidin divided into the three peptide segments synthesized (gold SA1, magenta SA2, and blue SA3). AlHx-K_6_ attachment point at Glu2 is italicized. Unnatural Cys34 and Cys77 are underlined. (C) Synthetic scheme with isolated yields for both l- and d-peptides at each purification step. NCL reactions were conducted with standard (1–2 mM) levels of peptide, 100 mM MPAA, and the following desulfurization was performed with 67 mM VA-044, 133 mM reduced GSH, and 200 mM TCEP. AlHx-K_6_ removal was achieved with 20 mM Pd(TPPTS)_4_ and 10 mM reduced glutathione in degassed ligation buffer, pH 8. See SI S8 and S10 for complete reaction details. The final protein was not RP-HPLC purified due to the high efficiency of the AlHx-K_6_ removal step. (D and E) RP-HPLC and MS overlays of unfolded recombinant (black), l- (orange), and d-streptavidin (green). As expected, all three proteins share the same retention time, ion peaks, and similar observed molecular weights (±0.9 g mol^−1^). Residual unprocessed Met can be observed in the recombinant SA MS sample.

Typically, the protein of interest is first synthesized using l-amino acids due to their higher quality and lower cost during troubleshooting and to validate bioactivity compared to a recombinant control. Once the synthetic l-protein is validated, the process is repeated using d-amino acids to yield the d-protein. Our lab has reported the synthesis of several d-proteins for use as targets in mirror-image phage display, which culminates in the identification of a d-peptide binder to the desired l-target.^23,32,33^ Throughout many CPS projects, we and others have identified major bottlenecks, such as poor peptide solubility and handling issues during intermediate steps (for an in-depth discussion see ref. 34–36). To overcome solubility challenges, we have developed semi-permanent ‘helping hands’ (HH) linkers that can be used on Lys (Ddap) and Glu (AlHx) residues to attach solubilizing cationic tags (*e.g.*, poly-Lys or poly-Arg) to the troublesome peptide during SPPS.^33,37–39^ HH linkers are stable to RP-HPLC and common CPS conditions and can be selectively removed from the full-length protein using either bis-α nucleophiles (Ddap), such as hydrazine or hydroxylamine, or a palladium catalyst (AlHx) to restore the native sequence. Here we report the total synthesis and folding of both l- and d-SA through a three-segment CPS strategy that was made possible by using the AlHx linker on the N-terminal segment (Fig. 2A).

In addition to our synthetic efforts, we measure a nearly 200-million-fold difference in affinity between the (−)-biotin and (+)-biotin interactions with SA using isothermal titration calorimetry (ITC). Furthermore, we determined two high-resolution crystal structures of each biotin enantiomer bound to recombinant SA to probe this dramatic difference in affinity. Together, these studies rigorously outline the total synthesis and folding of both l- and d-SA, assisted by our AlHx linker, and a new, efficient, and chiral-agnostic folding protocol. We define the affinity and structural differences between the matched and mismatched SA/biotin interactions, supporting the potential utility of the mirror-image SA/biotin system for overcoming biotin interference in diagnostic testing and utilizing its ultra-tight affinity in therapeutic platforms such as pretargeted radioimmunotherapy.

## Results and discussion

### AlHx-assisted total synthesis of l- and d-SA

*S. avidinii* expresses SA as a 159-residue polypeptide.^40^ Residues 1–12 and 140–159 are removed *via* proteolysis to generate the 127-residue ‘core’ SA (Fig. 2B).^41,42^ Longer SA sequences have been shown to be less stable and aggregation-prone, therefore we chose to synthesize the core 127-residue sequence, which also matches commercially available recombinant SA.^41^ Informed by Automated Ligator (Aligator), our in-house program to predict optimal synthetic strategies,^43^ we split SA into three peptide segments (Fig. 2B and C), SA1 (33 residues), SA2 (43 residues), and SA3 (51 residues), that we predicted could be easily synthesized and purified to homogeneity. Because SA does not have any natural Cys, we substituted Ala34 and Ala76 with Cys34 and Cys76, both of which are adjacent to Ser residues that make desirable thioesters for NCL.^44^ After the respective ligations, Cys34 and Cys76 can be converted to Ala *via* metal-free, radical-mediated desulfurization to regenerate the native sequence.^28^

The use of pseudoproline dipeptides^45^ and Fmoc-(2,4,6-trimethoxybenzyl)-Gly^46^ (Tmb)Gly to prevent on-resin aggregation and aspartimide formation enabled us to synthesize l-SA2 and l-SA3 in good quality, and both were purified *via* RP-HPLC (29% and 31% isolated yield, respectively, SI Section 7, Fig. S9 and S10). Our initial attempts to synthesize l-SA1 failed due to peptide insolubility in both RP-HPLC buffer (50–90% acetonitrile with water +0.1% trifluoracetic acid) and high levels of denaturants (6 M GdmHCl) (SI Section 7, Fig. S8). Although SA1 does not contain any cationic residues that aid solubility, it does have two Glu residues and a free N-terminus that could be suitable HH sites. We chose to use our pre-made Fmoc-l-Glu(AlHx-Dde)-OH amino acid building block, which can be incorporated directly *via* standard Fmoc-SPPS and deprotected on-resin with 5% hydrazine in DMF to yield an amine,^37^ which can be functionalized with a Lys6 tag through SPPS that enhances peptide solubility and handling. Using l-Glu(AlHx-Dde) at Glu2 we were able to synthesize l-SA1 and purify it to homogeneity *via* RP-HPLC (25% isolated yield, SI Section 7, Fig. S8).

With all three purified peptide segments, we began by ligating l-SA1(AlHx-K_6_) to l-SA2 under standard NCL conditions (100 mM MPAA) for 2 h (SI Section 8, Fig. S11). Following NCL, an overnight dialysis against ligation buffer (6 M GdmHCl, 100 mM phosphate, pH 7) with 5 mM tris(2-carboxyethyl)phosphine (TCEP) to remove the mercaptophenylacetic acid (MPAA) was conducted for one-pot metal-free radical desulfurization (67 mM VA-044, 133 mM reduced GSH, and 200 mM TCEP) at Cys34 to restore native Ala34 in 4 h at 50 °C. Although ∼20% Cys remained after 4 h, we reasoned that this would be removed in the following desulfurization after the final ligation. Longer desulfurization led to an accumulation of side-products and peptide degradation that complicated RP-HPLC purification of the ligated product (data not shown).^47,48^ Additionally, initial attempts at a global desulfurization of l-SA1-3(AlHx-K_6_) yielded incomplete desulfurization after extended reaction time (data not shown). Due to the difficulty of separating incomplete desulfurized peptide from the desired product, we reasoned that two separate NCL/desulfurizations would be a sufficient, albeit suboptimal, strategy to produce synthetic l- and d-SA for this study. While we believe the sluggish Cys34 desulfurization is influenced by the peptide sequence and local environment, another possibility is residual MPAA post-dialysis inhibiting the radical-mediated reaction.^49^ To mitigate the effects of residual aryl thiols, alternate thiols could be used, such as sodium 2-mercaptoethanesulfonate,^49^ methyl thioglycolate,^50^ or 2,2,2-trifluorethanethiol.^51^ There have also been some different approaches to MPAA removal or chemical inactivation that could be more effective than dialysis.^47,52,53^ More recently, there has been several new promising desulfurization strategies that could potentially overcome this slow reaction and will be investigated in future syntheses.^29,30,47,54–56^ Purified and desulfurized l-SA1-2(AlHx-K_6_) (25 mg, 38% isolated yield over two steps SI Section 8, Fig. S12), was ligated to l-SA3 (SI Section 8, Fig. S13) followed by another overnight dialysis step against ligation buffer with 5 mM TCEP. Next, desulfurized l-SA1-3(AlHx-K_6_) was obtained in 90 min using the same metal-free desulfurization conditions described above, efficiently restoring Ala76 (as well as removing residual Cys at Ala34). Desulfurized l-SA1-3(AlHx-K_6_) was purified by RP-HPLC (12 mg, 41% isolated yield over two steps, SI Section 8, Fig. S14). Finally, the AlHx-K_6_ linker was removed with 20 mM Pd(TPPTS)_4_ and 10 mM reduced glutathione in 6 M GdmHCl, 100 mM phosphate, pH 8 at 37 °C for 45 min. We then dialyzed twice against ligation buffer with 100 mM DTT to remove any residual Pd species, followed by a final dialysis step into 6 M GdmHCl, 80 mM Tris, pH 8 (5 mg, 94% isolated yield after final dialysis step, SI Section 8, Fig. S15). Due to the high purity of synthetic l-SA, no additional RP-HPLC purification was necessary prior to folding (described below). Because of the small scale of this synthesis, we did not perform inductively coupled plasma mass spectrometry (ICP-MS), which has been used to detect residual metal levels in past CPS contexts.^57–59^ For future large-scale SA production that will be RP-HPLC purified and used for biological studies, this analysis will be done to confirm the removal of Pd.

With a verified synthetic route in hand, we then began the synthesis of d-SA. After synthesizing the necessary d-pseudoproline dipeptides (not commercially available) and Fmoc-d-Glu(AlHx-Dde)-OH (SI Sections 5, 6 and Fig. S1–S7), all three d-peptide segments were made with comparable crude quality and purified to homogeneity *via* RP-HPLC following the protocols described above (SI Section 9, Fig. S16–S18). The ligation between d-SA1(AlHx-K_6_) and d-SA2 proceeded without issue, and after overnight dialysis to remove MPAA, we desulfurized Cys34 to Ala34 with the same efficiency and purity as its l-counterpart (32 mg, 39% isolated yield, SI Section 10, Fig. S19 and S20). After ligating to d-SA3 and the subsequent desulfurization, d-SA1-3(AlHx-K_6_) was purified with 25% isolated yield over two steps (7 mg, SI Section 10, Fig. S21 and S22). The AlHx-K_6_ linker was removed *via* the Pd method, and d-SA was dialyzed against 100 mM DTT in ligation buffer twice followed by 6 M GdmHCl, 80 mM Tris, pH 8.0 without RP-HPLC purification (4.6 mg, 71% isolated yield after final dialysis step, SI Section 10, Fig. S23).

### Folding and biophysical characterization of synthetic SA

Our first attempts at folding using recombinant SA followed established dilution-based methods,^21,60^ which involved sequential dilution steps to lower the initial denaturant (6 M GdmHCl). We reconstituted lyophilized recombinant SA in 6 M GdmHCl, 80 mM Tris, pH 8.0 (60 μM), incubated at 85 °C for 45 min, and verified the complete unfolding of SA with circular dichroism (CD) at room temperature, mimicking the starting pre-folding condition for synthetic SA (SI Section 11, Fig. S24). In our hands, we observed significant protein precipitation with this method and were only able to achieve 22% yield (SI Section 11, Table S1), which prompted us to search for an improved folding protocol. We developed such a method using 4-hydroxyazobenzene-2-carboxylic acid (HABA), a commonly used SA-binding azo-based dye. SA binds HABA weakly (100 μM),^61^ which we hypothesized could aid in stabilizing the correct folded conformation, while still allowing for easy removal from the folded product. Importantly, HABA is achiral and could be used equivalently for both l- and d-SA.

**Fig. 3:**
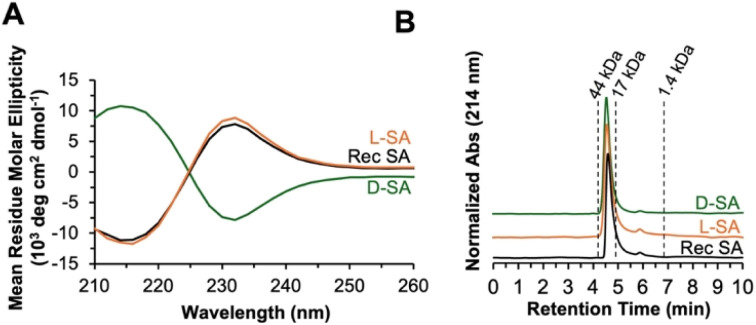
Biophysical characterization of recombinant, l- and d-synthetic SA. Biophysical characterization of recombinant (black), l- (orange), and d-SA (green) after folding. (A) CD spectrum overlay of all three proteins shows highly similar curves for recombinant and l-SA, while d-SA shows the inverted spectrum due to its mirror-image chirality. (B) Analytical SEC chromatography verifies the same oligomerization state of recombinant and both synthetic SA proteins. Molecular weight standards are shown as dotted lines corresponding to their retention times.

We first optimized our HABA-assisted folding method using unfolded recombinant SA. SA refolding was achieved by a six-fold drop-wise dilution into 80 mM Tris, pH 8.0 containing 7.5 molar equivalents of HABA (450 μM) with stirring for 30 min at room temperature. Dialysis against 80 mM Tris, pH 8.0 at 4 °C overnight removed GdmHCl and HABA (confirmed by A350 and A500 measurements). This method doubled the folding yield to 55% in comparison to the dilution-based approach. Encouraged by these results, we tried higher levels of HABA (75 molar equivalents) with recombinant SA (59% yield), l-SA (44% yield), and d-SA (34% yield), all of which were successfully folded as verified by CD (SI Section 11, Table S1). The l-SA CD spectrum was comparable to recombinant SA, and d-SA showed the mirror-image spectrum (Fig. 3A). We then verified the oligomeric state *via* size-exclusion chromatography (SEC), which confirmed that both l- and d-SA match recombinant SA (Fig. 3B). With folded l- and d-SA in hand, we next analyzed the binding affinity of biotin to the synthetic proteins through ITC.

### Isothermal titration calorimetry

As is inherent in ITC experiments with extremely high-affinity interactions such as the matched SA and biotin binding pair, Δ*H* and stoichiometry can be determined precisely, but K_D_ cannot (due to the sharp binding transition).^62^ For (+)-biotin/recombinant SA (Fig. S25), the measured enthalpy (Δ*H*) is −30 kcal mol^−1^ and stoichiometry (*N*) is 0.99 (per SA monomer) (see Table 1 for a complete list of values and SI Section 12). Using the reported SA K_D_ value (40 fM),^1^ we calculated the Δ*G* and entropic component of the interaction (Δ*G* = −18 kcal mol^−1^, −*T*Δ*S* = 12 kcal mol^−1^). This experiment was repeated with our synthetic l-SA and (+)-biotin (Fig. S27), and the measured Δ*H* of −30 kcal mol^−1^ with an *N* value of 0.96 agrees with the recombinant protein.

**Table 1:** ITC data for the biotin/SA interaction reported as the mean ± SD of duplicate measurements. *Indicates Δ*G* and −*T*Δ*S* values that were calculated using the measured Δ*H* and previously reported *K*_D_ value (not directly measured here)

Biotin enantiomer	Protein	*N* value (binding sites)	*K*_D_ (M)	Δ*G* (kcal mol^−1^)	Δ*H* (kcal mol^−1^)	−*T*Δ*S* (kcal mol^−1^)
**Matched Interactions**						
(+)-Biotin	Rec SA	0.99 ± 0.008	4 × 10^−14^*	−18*	−30 ± 0.12	12*
(+)-Biotin	l-SA	0.96 ± 0.007	4 × 10^−14^*	−18*	−30 ± 0.78	12*
(−)-Biotin	d-SA	0.98 ± 0.007	4 × 10^−14^*	−18*	−27 ± 0.055	8.5*

**Mismatched Interactions**						
(−)-Biotin	Rec SA	0.91 ± 0.017	8.0 ± 0.32 × 10^−6^	−7.0	−13 ± 0.095	6.0
(−)-Biotin	l-SA	0.84 ± 0.019	7.1 ± 0.84 × 10^−6^	−7.0	−12 ± 0.41	4.6
(+)-Biotin	d-SA	0.85 ± 0.041	6.9 ± 0.33 × 10^−6^	−7.0	−11.24 ± 0.72	4.2

Next, we titrated unnatural (−)-biotin (commercially available) into recombinant or synthetic l-SA (Table 1, Fig. S26 and S28). We found that there is an interaction between these molecules, and it was sufficiently weak to fit the *K*_D_ (8.0 and 7.0 μM, respectively). This mismatched pair had Δ*G*, Δ*H*, and −*T*Δ*S* values that were lower than the corresponding values for the matched binding pair (Δ*G* = −7.0, Δ*H* = −13, −*T*Δ*S* = 6.0 kcal mol^−1^ for recombinant SA with (−)-biotin and Δ*G* = −7.0, Δ*H* = −12, −*T*Δ*S* = 4.6 kcal mol^−1^ for l-SA with (−)-biotin). Titrating (−)-biotin into d-SA (Fig. S30) yields a measured Δ*H* value (−27 kcal mol^−1^) and stoichiometry (*N* = 0.98) similar to the matched, high-affinity interaction. Titrating (+)-biotin into d-SA (Fig. S29) (mismatched pair) resulted in a similar weak interaction with a *K*_D_ value of 6.9 μM. Because of the nearly 200-million-fold difference in binding between the matched and mismatched interaction, natural (+)-biotin at physiologic concentrations (even with supplementation) should not compete with (−)-biotin for binding to d-SA, making these interactions functionally orthogonal. While our results are consistent with the previously reported observation that there is no appreciable binding of the mismatched interaction at 4 nM,^21^ we provide a quantitative characterization of the expansive difference in affinities between the two interactions.

### X-ray crystal structures with recombinant SA

In hopes of structurally explaining the large, measured differences in ligand binding, we determined crystal structures of recombinant SA in complex with (+)- or (−)-biotin at very high resolutions (PDB: 9PUB – 0.95 Å and PDB: 9PUA – 0.94 Å, respectively) (Table S2). Both complexes were crystalized under identical, previously published conditions,^63^ thereby minimizing experimental differences between the two structures. SA forms a stable tetramer in solution^63^ and crystallized, as expected, in space group *I*222 with a half-tetramer (dimer) in the asymmetric unit (SI Section 13).

A previously defined set of 62 non-ligand binding core residues^63^ for each molecule in the streptavidin tetramer was overlapped closely on C-alpha coordinates (using the program lsqkab).^64^ We observe an rmsd of 0.007 Å^2^ when overlapped on 62 × 4 C-alpha atoms, indicating that there are no gross structural differences between SA bound to either ligand. Total C-alpha coordinates for the (+)- and (−)-biotin-bound structures also differed very little from the published SA-(+)-biotin structure, 3RY2 (rmsd 0.086 Å^2^ and 0.126 Å^2^, respectively). These small differences in atomic positions indicated nearly identical overall core structures and are on the order of overall coordinate error in the refined models, estimated to be 0.10 Å based on refinements in the Refmac5 software.^65^

**Fig. 4:**
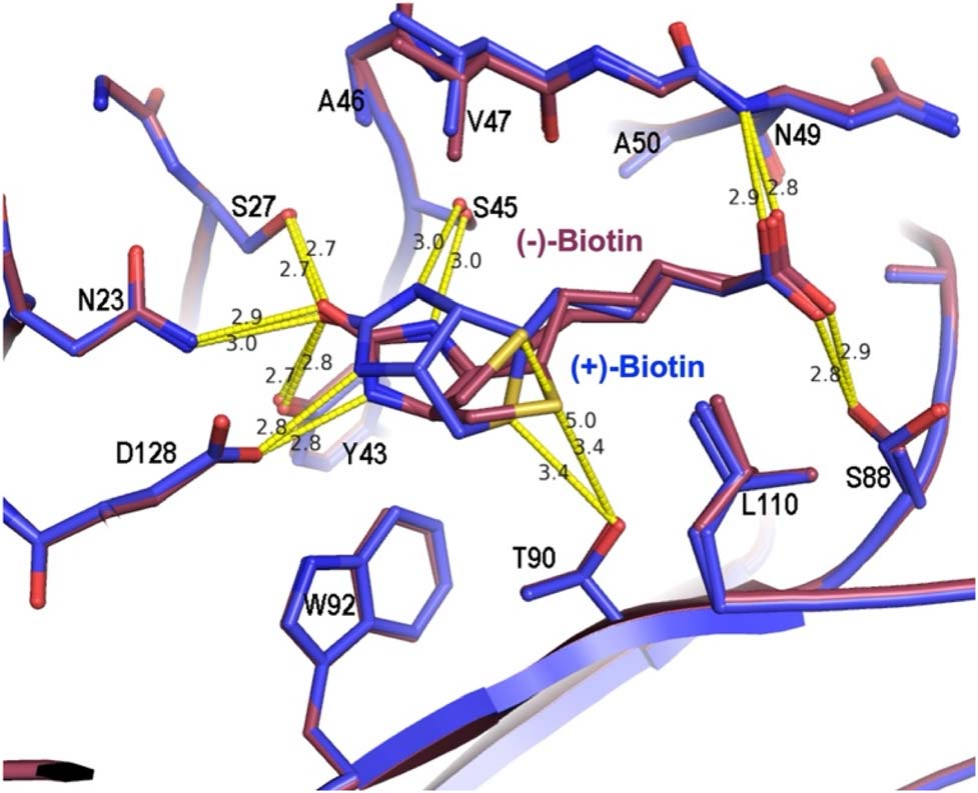
Structural analysis of recombinant SA with (+)- or (−)- biotin. Overlapped structures of recombinant SA bound to (+)-biotin (blue, 9PUB) or (−)-biotin (magenta, 9PUA). The biotin binding site is shown, including the two observed conformations of (−)-biotin (A and B). Prominent H-bonds are indicated with dotted lines and distances in Å.

Both enantiomers of biotin occupy similar positions and orientations in the binding site of their respective structures (Fig. 4). The most striking difference between (+)- and (−)- biotin binding geometry is the shift in the bicyclic ring system from concave ((+)-biotin) to convex ((−)-biotin) caused by inversion of all three stereocenters. In addition, examination of electron density maps prompted us to model (−)-biotin in two alternate conformations with equal relative occupancy to account for obvious flexibility of the ligand (Fig. 4), suggesting that (−)-biotin is less tightly constrained by interactions with SA, likely due to the lack of optimized binding. The increased flexibility of (−)-biotin in the ligand-binding site is consistent with the reduced entropic penalty measured by ITC (Table 1).

As previously demonstrated through mutational studies and computational predictions, hydrogen bonds play a key role in the biotin-SA interaction.^66,67^ All hydrogen-bonding interactions with the ligand ureido ring and, at the opposite end of the molecule, the carboxylic acid side chain are essentially unchanged between the two ligands. In the middle of the biotin molecule, the change in pucker of the heterocycle likely alters lipophilic interactions with residues immediately surrounding the ligand that presumably contribute significantly to the reduced binding affinity of (−)-biotin. This change in ring shape substantially alters the sulfur-centered hydrogen bond between Thr90 and the sulfur of the tetrahydrothiophene ring.

The sulfur of (−)-biotin occupies two alternate positions, A and B, with ∼50% occupancy each, retaining hydrogen-bonding to Thr90 in only the A position. The hydrogen-bond distance between the sulfur in (−)-biotin, conformation A, and Thr90 is about the same (3.4 Å) as the interaction with (+)-biotin. In contrast, in conformation B, the distance is increased to 5.0 Å, which indicates a much weaker interaction. The change in angle between the conformation A sulfur and Thr90 (168° for (+)-biotin *vs.* 139° for (−)-biotin) may also impact the strength of this H-bond, although the angular dependence of these interactions has been difficult to assess.^68,69^ Furthermore, the *K*_D_ of desthiobiotin, a biotin variant lacking the sulfur atom, is 10 pM, ∼3 orders of magnitude weaker than the native biotin interaction.^1,69^ Thus, differences in the Thr90 to biotin sulfur hydrogen bond likely partially contribute to the loss of affinity for (−)-biotin, although this is unlikely to be the only cause. In summary, while we were able to obtain high-resolution crystals of recombinant SA with both enantiomers of biotin, these structures provided insufficient information to identify the main causes of the vast difference in affinity between these two ligands.

## Conclusions

SA has been a vital biotechnology tool for decades, but its therapeutic and diagnostic use is limited by immunogenicity and endogenous biotin interference. A mirror-image SA/biotin system could provide an ideal solution to these challenges, though it has been unclear whether natural (+)-biotin would exhibit significant cross (mismatched) binding. Although cross-reactivity (or lack thereof) has been observed in the past with protein-ligand interactions, the degree of enantiospecificity is usually underexplored, as demonstrated here with SA/biotin.^21^ To evaluate the feasibility of this new technology, we synthesized d-SA and thoroughly characterized the affinity of the mismatched interaction. We outline a straightforward, Glu(AlHx)-assisted total synthesis of both l- and d-SA as well as a novel, high-efficiency folding protocol to generate functional synthetic SA tetramers of both chiralities. We show that the mismatched interactions between l-SA and (−)-biotin or d-SA and (+)-biotin are nearly 200-million-fold weaker than the matched interactions. This dramatic difference in affinity means that these two systems will be functionally orthogonal under physiologic conditions (up to ∼50–200 nM of (+)-biotin in those who supplement), a key property needed to enable maximum selectivity and impact in biological systems.

Furthermore, our crystallographic data gives some insight into the difference in affinity between the two biotin enantiomers. These high-resolution structures show the rigidity of the SA binding site, as it adapts to (−)-biotin with only minor structural differences, presumably reflecting the presentation of an optimal surface for binding of (+)-biotin. The two ligands bind in similar orientations, providing limited insight into the basis for the dramatic difference in affinity. One important difference is that (−)-biotin binds in a mixture of two conformations, likely reflecting lack of complementarity for the enantiomeric ligand. Additionally, the sulfur-containing hydrogen-bond between the tetrahydrothiophene ring of biotin and Thr90 is partly lost as only one conformation of (−)-biotin, representing ∼50% occupancy, participates in this interaction. We postulate that additional subtle changes in numerous hydrophobic interactions also contribute to the large difference in affinity between the two ligands, although these changes are non-obvious and difficult to characterize. We hope that these high-resolution data and observations will inform future computational prediction and design of high-affinity binding pairs, as well as lead to the development of a mirror-image SA/biotin system for biotechnology, diagnostic, and therapeutic applications.

## Author contributions

R. J. G. and P. C. S. W. conducted experiments, developed and designed methods, analyzed the data, and wrote the manuscript. S. R. E. D. conducted experiments, developed methods, and analyzed the data. P. S. conducted experiments. F. G. W. conducted experiments, analyzed the data, and helped write the manuscript. M. S. K. and C. P. H. conceived and supervised the research and acquired funding. All authors contributed to manuscript revisions and editing.

## Conflicts of interest

M. S. K. and S. R. E. D. are inventors on a WIPO PCT application WO2023220761A2 submitted by the University of Utah that covers the use of mirror-image streptavidin for targeted therapeutics.

## Supplementary Material

SC-OLF-D5SC06380A-s001

## Data Availability

The data supporting this article have been included as part of the supplementary information (SI). Crystallographic data for the streptavidin structures have been deposited at the PBD under 9PUA and 9PUB. Supplementary information is available. See DOI: https://doi.org/10.1039/d5sc06380a.
