# Supplementary material for: Protein chirality as a determinant of ligand affinity: insights from l- and d-streptavidin
Source: Chem Sci. 2025 Oct 28;16(48):23342–50. doi: 10.1039/d5sc06380a (PMC12593833; doi:10.1039/d5sc06380a)
Supplement: SC-016-D5SC06380A-s001 [file SC-016-D5SC06380A-s001.pdf]

Supplementary Information (SI) for Chemical Science.
This journal is © The Royal Society of Chemistry 2025

Supplementary Information

Protein Chirality as a Determinant of Ligand Affinity: Insights from L- and D-Streptavidin

Riley J. Giesler, Peter C.S. Woodham, Steven R. E. Draper,[†] Paul Spaltenstein,[§] Frank G. Whitby, Christopher P. Hill, and Michael S. Kay*

Department of Biochemistry, University of Utah,15 North Medical Drive East, Room 4100, Salt Lake City, UT, 84112, United States

[†]Current affiliation: Proteomics Core, Mayo Clinic, Rochester, MN, 55905, United States

[§]Current affiliation: Aliri Bioanalysis Salt Lake City, UT 84106, United States

*Corresponding Author: kay@biochem.utah.edu

Supplementary Information Contents:

1 Materials

2 General Methods for Peptide Synthesis and Cleavage

3 General Methods for RP-HPLC, LC/MS and SEC Analysis

4 General Methods for NCL, Desulfurization, and AlHx Removal

5 Synthesis and Characterization of Fmoc-(D)-psuedoprolines

6 Synthesis and Characterization of Fmoc-(D)-Glu(AlHx)-OH

7 Synthesis of (L)-Streptavidin – Initial Peptide Segments

8 Synthesis of (L)-Streptavidin – Ligation products and Full-Length Protein

9 Synthesis of (D)-Streptavidin – Initial Peptide Segments

10 Synthesis of (D)-Streptavidin – Ligation products and Full-Length Protein

11 Folding of Synthetic (L)- and (D)-Streptavidin and Circular Dichorism Analysis

12 Characterization of Synthetic (L)- and (D)-Streptavidin-biotin binding via ITC

13 Analysis of Streptavidin-biotin interaction through X-ray crystallography

14 Supplementary Figures, Schemes, and Tables

Supplementary Figures, Schemes, and Tables:

Figure S1 – Synthetic scheme and characterization of Fmoc-Gly-D-Thr($\Psi^{Me,Me}$pro)-OH

Figure S2 – Synthetic scheme and characterization of Fmoc-D-Val-D-Thr($\Psi^{Me,Me}$pro)-OH

Figure S3 – Synthetic scheme and characterization of Fmoc-D-Asp(tBu)-D-Ser($\Psi^{Me,Me}$pro)-OH

Figure S4 – Synthetic scheme and characterization of Fmoc-D-Leu-D-Thr($\Psi^{Me,Me}$pro)-OH

Figure S5 – Synthetic scheme and characterization of Fmoc-D-Lys(Boc)-D-Ser($\Psi^{Me,Me}$pro)-OH

Figure S6 – Synthetic scheme and characterization of Fmoc- D-Asp(tBu)-D-Thr($\Psi^{Me,Me}$pro)-OH

Figure S7 – Synthetic scheme and characterization of Fmoc-D-Glu(AlHx-Dde)-OH

Figure S8 – Characterization of L-SA1(AlHx-K_6) (1)

Figure S9 – Characterization of L-SA2 (2)

Figure S10 – Characterization of L-SA3 (3)

Figure S11 – NCL of L-SA1(AlHx-K_6) (1) and L-SA2 (2)

Figure S12 – Desulfurization of L-SA1-2(AlHx-K_6)_C34 (4)

Figure S13 – NCL of L-SA1-2(AlHx-K_6) (5) and L-SA3 (3)

Figure S14 – Desulfurization of L-SA1-3(AlHx-K_6)_C77 (6)

Figure S15 – Removal of AlHx-K_6 from L-SA1-3(AlHx-K_6) (7)

Figure S16 – Characterization of D-SA1(AlHx-K_6) (1*)

Figure S17 – Characterization of D-SA2 (2*)

Figure S18 – Characterization of D-SA3 (3*)

Figure S19 – NCL of D-SA1(AlHx-K_6) (1*) and D-SA2 (2*)

Figure S20 – Desulfurization of D-SA1-2(AlHx-K_6)_c34 (4*)

Figure S21 – NCL of D-SA1-2(AlHx-K_6) (5*) and D-SA3 (3*)

Figure S22 – Desulfurization of D-SA1-3(AlHx-K_6)_c77 (6*)

Figure S23 – Removal of AlHx-k_6 from D-SA1-3(AlHx-K_6) (7*)

Figure S24 – CD spectrum of unfolded and refolded recombinant SA

Figure S25 – ITC data for recombinant SA with (+)-biotin

Figure S26 – ITC data for recombinant SA with (-)-biotin

Figure S27 – ITC data for L-SA with (+)-biotin.

Figure S28 – ITC data for L-SA with (-)-biotin.

Figure S29 – ITC data for D-SA with (+)-biotin.

Figure S30 – ITC data for D-SA with (-)-biotin.

Figure S31 – Unbiased electron density for (-)-biotin.

Table S1 – Table of folding yields for Recombinant, L-, and D-SA.

Table S2 – Crystallography table for recombinant SA complexed with (+)- and (-)-biotin.

List of Peptide and Compounds Synthesized:

Peptide Name	Calculated Mol Weight (g/mol)	Observed Mol Weight (g/mol)
L-SA1(AlHx-K_6) **(1)**	4,288.6	4,289.1
L-SA2 **(2)**	4,630.8	4,631.9
L-SA3 **(3)**	5,429.8	5,428.9
L-SA1-2(AlHx-K_6)_C34 **(4)**	8,887.3	8,888.5
L-SA1-2(AlHx-K_6) **(5)**	8,855.4	8,856.9
L-SA1-3(AlHx-K_6)_C77 **(6)**	14,254.3	14,253.4
L-SA1-3(AlHx-K_6) **(7)**	14,222.0	14,220.8
L-SA1-3 **(8)**	13,271.3	13,269.5
D-SA1(AlHx-K_6) **(1*)**	4,288.6	4,289.1
D-SA2 **(2*)**	4,630.8	4,631.9
D-SA3 **(3*)**	5,429.8	5,428.9
D-SA1-2(AlHx-k_6)_c34 **(4*)**	8,887.3	8,888.5
D-SA1-2(AlHx-k_6) **(5*)**	8,855.4	8,856.9
D-SA1-3(AlHx-k_6)_c77 **(6*)**	14,254.3	14,253.4
D-SA1-3(AlHx-k_6) **(7*)**	14,222.0	14,220.8
D-SA1-3 **(8*)**	13,271.3	13,269.5

Compound Name	Calculated Mol Weight (g/mol)	Observed Mol Weight (g/mol)
Fmoc-Gly-D-Thr($\Psi^{Me,Me}$pro)-OH **(X1)**	438.1	439.1
Fmoc-D-Val-D-Thr($\Psi^{Me,Me}$pro)-OH **(X2)**	480.4	481.1
Fmoc-D-Asp(tBu)-D-Ser($\Psi^{Me,Me}$pro)-OH **(X3)**	538.2	539.1
Fmoc-D-Leu-D-Thr($\Psi^{Me,Me}$pro)-OH **(X4)**	494.8	495.2
Fmoc-D-Lys(Boc)-D-Ser($\Psi^{Me,Me}$pro)-OH **(X5)**	594.5	596.5
Fmoc-D-Asp(tBu)-D-Thr($\Psi^{Me,Me}$pro)-OH **(X6)**	552.5	553.2
Dde-Ahx-OH **(X7)**	295.2	296.1
Dde-Ahx-OAll-OH **(X8)**	365.2	365.6
Fmoc-D-Glu(AlHx-Dde)-OH **(X9)**	716.6	717.8

S1 Materials:

Solid-Phase Peptide Synthesis (SPPS)

2-chlorotrityl chloride resin (0.77 mmol/g) was purchased from ChemPep. Fmoc-L-Ala-OH, Fmoc-L-Cys(Trt)-OH, Fmoc-L-Asp(OtBu)-OH, Fmoc-L-Glu(OtBu)-OH, Fmoc-L-Phe-OH, Fmoc-Gly-OH, Fmoc-L-His(Trt)-OH, Fmoc-L-Ile-OH, Fmoc-L-Lys(Boc)-OH, Fmoc-L-Leu-OH, Fmoc- L-Asn(Trt)-OH, Fmoc-L-Pro-OH, Fmoc-L-Gln(Trt)-OH, Fmoc-L-Arg(Pbf)-OH, Fmoc-L-Ser(tBu)-OH, Fmoc-L-Thr(tBu)-OH, Fmoc-L-Val- OH, Fmoc-L-Trp(Boc)-OH, and Fmoc-L-Tyr(tBu)-OH were purchased from Gyros Protein Technologies and Iris Biotech GmbH. Boc-L-Ala-OH and Boc-L-Cys(Trt)-OH were purchased from Sigma Aldrich. Fmoc-Leu-Thr(Ψ*Me,Me*pro)-OH, Fmoc-Val-Thr(Ψ*Me,Me*pro)-OH, Fmoc-Gly-Thr(Ψ*Me,Me*pro)-OH, Fmoc-Asp-Thr(Ψ*Me,Me*pro)-OH, Fmoc-Asp- Ser(Ψ*Me,Me*pro)-OH, Fmoc-Lys(Boc)-Ser(Ψ*Me,Me*pro)-OH, and Fmoc-(2,4,6-trimethoxybenzyl)-glycine-OH were purchased from Iris Biotech GmbH.

Fmoc-D-Asp(OtBu)-OH, Fmoc-D-Glu(OtBu)-OH, Fmoc-D-His(Trt)-OH, Fmoc-D-Lys(Boc)-OH, Fmoc- D-Asn(Trt)-OH, Fmoc-D-Arg(Pbf)-OH, Fmoc-D-Ser(tBu)-OH, Fmoc-D-Thr(tBu)-OH, Fmoc-D-Val- OH, Fmoc-D-Trp(Boc)-OH, and Fmoc-D-Tyr(tBu)-OH were purchased from GL Biochem. Fmoc-D-Ala-OH, Fmoc-D-Cys(Trt)-OH, and Fmoc-D-Ile-OH were purchased from Chempep. Fmoc-D-Phe-OH and Fmoc-D-Pro-OH were purchased from CBL Patras. Fmoc-D-Leu-OH and Fmoc-D-Gln(Trt)-OH were purchased from AA blocks. Boc-D-Ala-OH was purchased from Sigma Aldrich and Boc-D-Cys(Trt)-OH was purchased from AAPPtec.

Anhydrous hydrazine (98%), *N,N*-diisopropylethylamine (DIPEA, ReagentPlus grade), piperidine (ReagentPlus grade), *N*-methylmorpholine (NMM, ReagentPlus grade) were purchased from Sigma Aldrich. Dimethylformamide (DMF, ACS grade), dichloromethane (DCM, ACS grade), N-methylpyrrolidone (NMP, ≥99.8%), and methanol (MeOH, ACS grade) were purchased from Fisher Scientific. 1- [Bis(dimethylamino)methylene]-1*H*-1,2,3-triazolo[4,5-*b*]pyridinium 3-oxid hexafluorophosphate (HATU, 99%) was purchased from Oakwood Chemical. 9-Fluorenylmethyl carbazate was purchased from Chem-Impex.

Amino Acid Synthesis

H-D-Thr-OH, H-D-Ser-OH, Fmoc-D-Glu-OtBu, (*E*)-2-Butene-1,4-diol, 2-acetyldimedone, and *N*-Hydroxysuccinimide (NHS) were purchased from Ambeed chemicals. *N*-(3-Dimethylaminopropyl)-*N'*-ethylcarbodiimide hydrochloride (EDCI) was purchased from AAPPtec. pyridinium p-toluenesulfonate (PPTS) and dimethoxypropane (2,2-DMP) were purchased from Sigma Aldrich. Tetrahydrofuran (THF) (≥99.9%) and ethyl acetate(EtOAc) (ACS grade) were purchased from Fisher Scientific. Fmoc-L-Glu((*E*)-4-hydroxybut-2-en-1-yl 6-((1-(Dde)amino)hexanoate))-OH (Fmoc-L-Glu(AlHx-Dde)-OH) was synthesized as described in the established protocols.[1, 2]

Peptide Cleavage and Precipitation

Trifluoroacetic acid (TFA, peptide synthesis grade) and anhydrous ethyl ether (ACS grade) were purchased from Fisher Scientific. Triisopropylsilane (TIS, 98%) was purchased from Sigma Aldrich. 1,2-ethanedithiol (EDT, 95%) was purchased from Acros Organics.

RP-HPLC and LC-MS

Trifluoroacetic acid (TFA, HPLC grade) was purchased from Alfa Aesar. Acetonitrile (MeCN, HPLC grade), 0.1% formic acid in water (Optima LC-MS grade), and 0.1% formic acid in acetonitrile (Optima LC-MS grade) were purchased from Fisher Scientific. SEC standards (lyophilized mix of thyroglobulin, bovine γ-globulin, chicken ovalbumin, equine myoglobin, and vit B12, MW 1,350–670,000, pI 4.5–6.9) were purchased from biorad.

Ligation, Desulfurization, Dialysis, AlHx Linker Removal, and Folding

Guanidine hydrochloride (GdmHCl, ≥99.5%) was purchased from Thermo Scientific. Sodium phosphate dibasic heptahydrate (ACS grade) was purchased from AMRESCO. Sodium phosphate monobasic (ACS grade), hydrochloric acid (HCl, ACS plus grade), 4-mercaptophenylacetic acid (MPAA, 97%), and sodium nitrite (NaNO2, ACS grade) were purchased from Fisher Scientific. Sodium hydroxide (NaOH, ACS grade), hydrochloric acid (HCl, ACS grade), and acetic acid (>99.8%) were purchased from Acros Organics. Tris (2-carboxyethyl) phosphine hydrochloride (TCEP-HCl, ≥99%) was purchased from Hampton Research. VA-044 was purchased from Wako. L-glutathione reduced (GSH, ≥98%), palladium(II) acetate (Pd(OAc)2, 98%), and triphenylphosphine- 3,3',3"-trisulfonic acid trisodium salt (TPPTS, ≥95%) were purchased from Sigma Aldrich. Dithiothreitol (DTT, ≥99%) was purchased from Gold Biotechnology. Dialysis slide-a-lyzer cassettes were purchased from Fisher Scientific. Tris HCl and 4'-hydroxyazobenzene-2-carboxylic acid (HABA) were purchased from Thermo Scientific.

ITC and Crystallography

Recombinant streptavidin and D-biotin were purchased from Thermo Scientific. L-biotin was purchased from MedChemExpress. EDTA and $CaCl_2$ standards for ITC calibration were provided by Malvern. Ammonium sulfate and Sodium acetate were purchased from Thermo Scientific.

S2 General methods for Peptide Synthesis and Cleavage:

Automated Peptide Synthesis

All peptides were synthesized on Prelude X or PurePep Chorus instruments (Gyros Protein Technologies) using Fmoc SPPS. All deprotection and coupling cycles were performed with nitrogen bubbling, and gentle shaking was also used for coupling steps. All cycles were performed at room temperature (RT) unless otherwise specified.

For 25 µmol scale syntheses, deprotection cycles consisted of two consecutive 2 min treatments of 2 mL 20% PIP in DMF, followed by three 30 s washes of 2 mL DMF. Coupling cycles consisted of 25 min treatment with a solution of 0.65 mL 200 mM amino acid in DMF, 0.65 mL 195 mM HATU in DMF, and 0.5 mL 600 mM NMM in DMF, followed by three 30 s washes of 2 mL DMF.

For 50 µmol scale syntheses, deprotection cycles consisted of two consecutive 2 min treatments of 4 mL 20% PIP in DMF, followed by three 30 s washes of 4 mL DMF. Coupling cycles consisted of 25 min treatment with a solution of 1.4 mL 200 mM amino acid in DMF, 1.4 mL 195 mM HATU in DMF, and 1 mL 600 mM NMM in DMF, followed by three 30 s washes of 4 mL DMF.

Washing, Swelling, and Mixing of Resins

For 25 µmol scale syntheses, resins were washed and swelled with approximately 3 mL of specified solvent. The wash volumes for all other synthesis scales are indicated in the relevant sections below. All swelling and manual coupling/deprotection steps were mixed on a rotisserie at RT, unless otherwise specified.

Preparation of Peptide Resins

To prepare **C-terminal acid** peptides at 25 µmol scale, 150 mg 2-chlorotrityl chloride resin (0.77 mmol/g) was weighed into a 6 mL SPPS tube. The resin was washed three times with DMF, followed by three DCM washes. In order to load the first amino acid onto the resin, 0.03 mmol of the Fmoc-protected amino acid was dissolved in 1 mL of a 1:1 DMF/DCM mixture. 0.15 mmol DIPEA (26 µL) was then mixed into the amino acid solution. The entire amino acid and DIPEA solution was added to the 2-chlorotrityl chloride resin, and the coupling reaction was rotated for 1 h. The resin was then washed three times with DCM. Unreacted 2-chlorotrityl chloride was capped by repeatedly washing the resin with a 17:2:1 DCM:MeOH:DIPEA mixture (~20 mL total used). Once the capping reaction was complete, the resin was washed three times with DCM, followed by three DMF washes. Finally, the resin was transferred to the instrument for automated SPPS. This procedure generates resin with a loading density similar to Tentagel R RAM resin (approximately 0.2 mmol/g). See "*Determining Resin Loading Density using Fmoc Absorbance"* for more on loading density. The same protocol was followed for 50 µmol scale resin preparation with amounts (mg and mL) adjusted accordingly.

To synthesize **C-terminal peptide-NHNH$_2$** at 25 µmol scale, Fmoc-hydrazine on 2-chlorotrityl chloride resin was prepared based on an established protocol,[3] with several modifications. 150 mg of 2-chlorotrityl chloride resin (0.77 mmol/g) was weighed into a 6 mL SPPS tube. The resin was washed three times with DCM. 1.5 mL DCM was then added to the resin, and the resin was allowed to swell for 10 min at 4 °C. 30.6 µmol Fmoc-hydrazine (7.8 mg) was dissolved in a mixture of 1.5 mL DMF, followed by addition of 266 µL DIPEA. Once the 2-chlorotrityl chloride resin was finished swelling, the Fmoc-hydrazine and DIPEA solution was added to the resin slurry at 4 °C. The resin slurry was then placed on a rotisserie at RT, and the coupling reaction was rotated for 2 h. Once the coupling reaction was finished, 30 µL MeOH was added to the resin (without draining the SPPS tube) in order to cap unreacted 2-chlorotrityl chloride (rotated for 10 min). The resin was then washed three times with DMF, followed by three DCM washes. The resin was then swelled in a 1:1 DMF/DCM mixture for ≥ 10 min and transferred to the instrument for automated SPPS. This procedure generates resin with a loading density similar to Tentagel R RAM resin (approximately 0.2 mmol/g). See "*Determining Resin Loading Density using Fmoc Absorbance"* for more on loading density. The same protocol was followed for 50 µmol scale resin preparation with amounts (mg and mL) adjusted accordingly.

Determining Resin Loading Density using Fmoc Absorbance

The resin loading density was determined by Fmoc removal and subsequent Fmoc-piperidine adduct absorbance. To do so, the resin (25 µmol scale) was treated with 4 mL of 20% piperidine in DMF with mixing for 20 min. The Fmoc-piperidine solution was collected and diluted 100x with 20% piperidine in DMF. Triplicate Fmoc absorbance measurements were taken at 280 nm using a NanoDrop One^C instrument (Thermo Scientific) blanked with 20% piperidine in DMF. The average A_{280} was used to calculate the Fmoc concentration using an A_{280} extinction coefficient of 5680 $M^{-1}cm^{-1}$. From there, the loading density can be calculated as mmol/g.

Synthesis of Peptide with Lys$_6$-Functionalized Glu HH

Glu(AlHx) modified peptides were synthesized with an N-terminal Boc-protected residue to allow for site-specific modification of the AlHx linker. For 25 µmol scale Glu(AlHx-Dde) deprotection, peptide resin was treated with 300 µL allyl alcohol and 1.7 mL 5% hydrazine in DMF for three consecutive 5 min treatments, with mixing. Following the hydrazine treatment, the resin was washed six times with DMF. The resin was returned to the automated synthesizer and the Lys_6 tag was added with standard conditions described in '*Automated Peptide Synthesis.*'

Cleavage and Peptide Precipitation:

25 µmol scale peptide cleavage was accomplished with a 3 h treatment of 4 mL TFA containing 2.5% water and 2.5% TIS, with mixing on a rotisserie at RT For peptides containing Cys(Trt), 2.5% EDT was added to the TFA cocktail. After cleavage, the TFA solution was added to ~36 mL ice-cold ethyl ether, shaken thoroughly, and placed at -20 °C for ≥ 30 min, in order to precipitate the crude peptide. The solution was then centrifuged at 4,700 *g*, 4 °C for ≥ 10 min. The resulting supernatant was decanted, and crude peptide pellets were washed twice with ~20 mL ice-cold ethyl ether. The crude peptide pellets were dried in a vacuum desiccator overnight.

Preparation of Crude Peptides for Analytical and Preparative RP-HPLC and LC-MS:

25 µmol scale crude peptides were dissolved in 10-20% MeCN 0.1% TFA (~20-40 mL total volume) and were vortexed and sonicated to dissolve as much material as possible, followed by centrifugation at 4,700 *g*, 4 °C for 10 min prior to analytical RP-HPLC, preparative RP-HPLC, and/or LC-MS. Smaller and larger scale sample preparations were conducted by adjusting the amount of crude peptide and solvent adequately.

S3 General Method for RP-HPLC, LC/MS, and SEC Analysis:

Analytical LC-MS Methods:

0.1% formic acid in water (Buffer A) and 0.1% formic acid in MeCN (Buffer B) were used as mobile phases for LC-MS analyses. Mass spectra were obtained on an Agilent 6120 single-quadrupole mass spectrometer in fast scan/positive ion mode with an Agilent 1260 Infinity II front-end. UV data were collected using the Agilent 1260 Infinity II diode array detector (200-600 nm). Unless otherwise noted, observed masses were calculated using the charge states from averaged scans across the major ion signal and corresponding UV peak. Calculated and observed masses are presented as average mass. LC-MS methods are described below:

- **LC-MS Method A:** Phenomenex Aeris Widepore 3.6 µm C4 (200 Å, 2.1 x 50 mm) 50 °C; gradient: 0-1 min 5% B, 1-8 min 5-90% B, 8-8.1 min 90-5% B, 8.1-10 min 5% B; flow rate: 0-10 min 0.5 mL/min; scan range: 100-2,000 *m/z*; voltage: 50 V
- **LC-MS Method B:** Phenomenex Aeris Widepore 3.6 µm C4 (200 Å, 2.1 x 50 mm) 50 °C; gradient: 0-1 min 5% B, 1-16 min 5-50% B, 16-16.1 min 50-5% B, 16.1-18 min 5% B; flow rate: 0-18 min 0.5 mL/min; scan range: 100-2,000 *m/z*; voltage: 50 V
- **LC-MS Method C:** Phenomenex Aeris Widepore 3.6 µm C4 (200 Å, 2.1 x 50 mm) 50 °C; gradient: 0-1 min 10% B, 1-16 min 10-40% B, 16-16.1 min 40-5% B, 16.1-18 min 5% B; flow rate: 0-18 min 0.5 mL/min; scan range: 100-2,000 *m/z*; voltage: 50 V
- **LC-MS Method D:** Agilent Poroshell 2.7 µm EC-C18 (120 Å, 4.6 x 50 mm); 50 °C; gradient: 0-1 min 15% B, 1-11 min 15-95% B, 11-11.1 min 95-5% B, 11.1-12 min 5% B; flow rate: 0-11.1 min 0.8 mL/min, 11.1-12 min 0.8-1.0 mL/min, 8.1-10 min; scan range: 100-2,000 *m/z*; voltage: 90 V

Analytical SEC Method:

150 mM NaCl, 50 mM phos in water, pH 7.5 was used as mobile phase for SEC analysis. Spectra were obtained with an Agilent 1260 Infinity II. UV data were collected using the Agilent 1260 Infinity II diode array detector (200-600 nm). SEC standards (lyophilized mix of thyroglobulin, bovine γ-globulin, chicken ovalbumin, equine myoglobin, and vit B12, MW 1,350–670,000, pI 4.5–6.9) were run on the same day as the samples and their retention time was plotted to mark relative size. The method used is described below:

- **SEC Method A:** Phenomenex Yarra SEC X150 1.8 μm (90 Å, 4.6 x 150 mm); 30°C gradient: 0-10 min 100% 150 mM NaCl, 50 mM phos in water, pH 7.5; flow rate: 0.35 mL/min

Analytical RP-HPLC Methods:

0.1% TFA in water (Buffer A) and 0.1% TFA in 90% MeCN (Buffer B) were used as mobile phases for analytical RP-HPLC analyses. Traces were collected on an Agilent 1260 Infinity II instrument at A214. Analytical RP-HPLC methods are described below:

- **Analytical Method A:** Phenomenex Jupiter 4 μm C12 (90 Å, 4.6 x 150 mm); 40°C; gradient: 0-2 min 10% B, 2-27 min 10-90% B, 27-30 min 90% B, 30-30.1 min 90%-10 B, 30.1-34 min 10% B; flow rate: 1.0 mL/min
- **Analytical Method B:** Phenomenex Jupiter 4 μm C12 (90 Å, 4.6 x 150 mm); 50°C; gradient: 0-2 min 10% B, 2-27 min 10-90% B, 27-30 min 90% B, 30-30.1 min 90%-10 B, 30.1-34 min 10% B; flow rate: 1.0 mL/min
- **Analytical Method C:** Phenomenex Aeris Widepore 3.6 μm C4 (200 Å 4.6 x 150 mm); 50°C; gradient: 0-2 min 10% B, 2-27 min 10-90% B, 27-30 min 90% B, 30-30.1 min 90-10% B, 30.1-34 min 10% B; flow rate: 1.0 mL/min
- **Analytical Method D:** Phenomenex Jupiter 5 μm C4 (300 Å, 4.6 x 150 mm); 50°C; gradient: 0-1 min 10% B, 1-26 min 10-50% B, 26-26.1 min 50-90% B, 26.1-27.1 min 90% B, 27.1-27.2 min 90-10% B, 27.2-29 min 10% B; flow rate: 1.0 mL/min
-

Preparative RP-HPLC Methods:

0.1% TFA in water (Buffer A) and 0.1% TFA in 90% MeCN (Buffer B) were used as mobile phases for preparative RP-HPLC purifications. Peptide purifications were performed on an Agilent 1260 Infinity LC system semi-preparative or Agilent 1260 Infinity II preparative system. Fractions collected during preparative RP-HPLC purifications were analyzed by LC-MS to assess purity. All pure fractions were pooled and lyophilized to obtain dry, pure peptide which was then analyzed by analytical RP-HPLC and LC-MS. RP-HPLC purification methods are described below:

- **Purification Method A:** Phenomenex Jupiter 4 μm C12 (90 Å, 21.2 x 250 mm); RT; gradient: 0-4 min 10% B, 4-5 min 10-29% B, 5-30 min 29-34% B, 30-30.1 min 34-90% B, 30.1-34 min 90% B, 34-34.1 min 90-10% B, 34.1-38.5 min 10%B; flow rate: 15 mL/min

- **Purification Method B:** Phenomenex Jupiter 4 µm C12 (90 Å, 21.2 x 250 mm); RT; gradient: 0-4 min 10% B, 4-8 min 10-25% B, 8-33 min 25-29% B, 33-33.1 min 29-90% B, 33.1-37.1 min 90% B, 37.1-37.2 min 90-10% B, 37.2-42 min 10%B; flow rate: 20 mL/min
- **Purification Method C:** Phenomenex Jupiter 4 µm C12 (90 Å, 21.2 x 250 mm); RT; gradient: 0-4 min 10% B, 4-8 min 10-30% B, 8-33 min 30-36% B, 33-33.1 min 36-90% B, 33.1-37.1 min 90% B, 37.1-37.2 min 90-10% B, 37.2-42 min 10%B; flow rate: 20 mL/min
- **Purification Method D:** Phenomenex Jupiter 5 µm C4 (300 Å, 10 x 250 mm); 60°C; gradient: 0-5 min 10% B, 5-5.5 min 10-25% B, 5.5-30.5 min 25-55% B, 30.5-31 min 55-100% B, 31-35 min 100% B, 35-35.1 min 100-10% B, 35.1-40 min 10%B; flow rate: 4 mL/min
- **Purification Method E:** Phenomenex Jupiter 5 µm C4 (300 Å, 10 x 250 mm); 60°C; gradient: 0-5 min 10% B, 5-5.5 min 10-23% B, 5.5-35.5 min 23-55% B, 35.5-36 min 55-90% B, 36-40 min 90% B, 40-40.1 min 90-10% B, 40.1-44 min 10%B; flow rate: 4 mL/min

S4 General Method for NCL, Desulfurization, and AlHx Removal:

Peptide Hydrazide (Peptide-$NHNH_2$) Native Chemical Ligation (NCL):

To perform NCL of two peptide segments by peptide-$NHNH_2$ activation, the peptide-$NHNH_2$ was dissolved (~5-10 mM) and activated (conversion of hydrazide to acyl azide to thioester) in activation buffer (6 M GdmHCl, 100 mM phos, pH 3) for 20 min at -20 ºC by addition of freshly prepared 15 eq sodium nitrite ($NaNO_2$). Following activation, 50 eq of freshly prepared MPAA in ligation buffer (6 M GdmHCl, 100 mM phos, pH 6.5) was added to the peptide-N_3, and the final pH was adjusted to 6.8 to initiate thiolysis. Thiol-containing peptide was dissolved in ligation buffer to ~5-10 mM and added to the ligation reaction (1-1.5 eq of peptide). After 10 min, 15 mM TCEP in ligation buffer was added to the reaction and the reaction was left to rotate on a rotisserie at r.t until complete. Time points for the NCL reactions were taken by diluting an aliquot of reaction 1:1 with 200 mM TCEP in LC-MS-grade water (pH adjusted to 7.0 prior to addition). The peptide/TCEP mixture was then diluted 1:10 with Water + 0.1% TFA and the dilution was centrifuged at 18,000 g, r.t for 10 min prior to analytical RP-HPLC and/or LC-MS analyses. Upon completion (based on analytical RP-HPLC and LC-MS), reactions were treated with 1:1 with 200 mM TCEP in LC-MS-grade water (pH adjusted to 7.0 prior to addition). The reaction mixture was then dialyzed twice against ligation buffer (200X volume) to remove MPAA prior to desulfurization. The dialyzed reaction mixture was then checked by RP-HPLC and LC-MS to ensure no material loss and taken into the desulfurization reaction step.

Radical-Mediated, Metal-Free Desulfurization:

"Desulfurization buffer" (6 M GdmHCl, 100 mM phos, pH 6.5) was first sparged with argon gas for ≥ 20 min. 400 mM reduced glutathione, 200 mM VA-044 in desulfurization buffer was prepared, along with a solution of 600 mM TCEP in desulfurization buffer. Equal volumes of the peptide reaction, the VA-044/GSH solution, and TCEP solution were mixed and carefully pH adjusted to 6.5-6.8 using NaOH. The final concentrations were 67 mM VA-044, 133 mM GSH, 200 mM TCEP, and ~1 mM peptide. The desulfurization reaction was then covered with argon gas and mixed in an oil bath at 50 °C until completion. Time points were taken by first diluting 9 µL desulfurization reaction in 51 µL of water + 0.1% TFA. After vortexing, the dilution was

centrifuged at 18,000 g, RT for 10 min prior to analytical RP-HPLC and LC-MS analyses. The finished desulfurization reaction was purified by RP-HPLC.

AlHx Removal with Pd/TPPTS and Dialysis:

The AlHx removal was performed as previously reported.[1, 4] All solutions were degassed with argon for >10 min. Briefly, 350 mM GSH solution was prepared in degassed ligation buffer, 188 mM $Pd(OAc)_2$ was prepared in degassed DMF, and 850 mM TPPTS was prepared in degassed ddH_2O. The peptide was dissolved to ~1-2 mM in degassed ligation buffer. The $Pd(OAc)_2$ and TPPTS solutions were mixed in a 1:1 ratio, then added to the peptide to a final concentration of 20 mM $Pd(TPPTS)_4$. The reaction was briefly mixed with vortexing prior to adding GSH to a final concentration of 10 mM. The pH of the reaction was adjusted to 8.0, the reaction was degassed and placed on the rotisserie at 37 °C until complete. Time points of the reaction were taken by treating with a 1:1 ratio of 1 mM DTT (prepared in ddH_2O, pH 7.0) for 10 min at RT to quench the Pd. The time points were then acidified by diluting 1:10 into H_2O + 0.1% TFA followed by vortexing and centrifugation at 18,000 g, RT for 10 min prior to analytical RP-HPLC and LC-MS analyses. Once AlHx removal was complete, the reaction was dialyzed twice against ligation buffer with 100 mM DTT, pH 7.0 (200x volume). The reaction was then dialyzed into 6 M GdmHCl, 80 mM tris, pH 8 (200x volume).

S5 Synthesis and Characterization of Fmoc-D-psuedoprolines used:

Fmoc-Gly-D-Thr($\Psi^{Me,Me}$pro)-OH ***(X1) (Figure S1)***

A solution of Fmoc-Gly-OH (10 g, 33.67 mmol), and NHS (7.74 g, 67.34 mmol) were dissolved in 80 mL of 80%THF/DMF in an ice bath at 0°C. EDCI (12.9 g, 67.34 mmol) was added, the solution was warmed to RT and stirred for 4 hours. The reaction was concentrated under reduced pressure and diluted with EtOAc. The organic layer was washed 2x with H_2O, concentrated under reduced pressure, and resuspended in 100 mL of acetone. 12.5 g (168.35 mmol) of H-D-Thr-OH was dissolved in 30 mL of a 10% Na_2CO_3 (w/v) aqueous solution. The D-Thr solution was added to the acetone solution and stirred at RT overnight. The reaction was again concentrated under reduced pressure and diluted with H_2O. It was cooled to 0°C and slowly acidified with 2 N HCl to a pH <3. The dipeptide was extracted with EtOAc and washed with H_2O (3x) and brine (2x). The organic layer was dried with Na_2SO_4 and evaporated under reduced pressure. The crude dipeptide Fmoc-Gly-D-Thr-OH was lyophilized in 75% ACN/H_2O and used without further purification.

Crude Fmoc-Gly-D-Thr-OH (8 g, 20 mmol), 2,2-DMP (50.2 g, 200 mmol), and PPTS (1.04 g, 10 mmol) were dissolved in 200 mL of dry THF at RT. The RB flask was fitted with a reflux condenser and placed in an oil bath. The reaction was heated to reflux for 24 hours. The reaction was cooled to RT and the PPTS was neutralized with 700 µL of triethylamine. The THF was removed under reduced pressure and the reaction was diluted with EtOAc. The organic layer was washed 3x with H_2O. The EtOAc layer was dried with Na_2SO_4 and purified by reverse-phase flash chromatography with a H_2O/ACN mixture (10-100% ACN over 35 min) to afford Fmoc-Gly-D-Thr($\Psi^{Me,Me}$pro)-OH **(X1)** as a white fluffy powder (4.1 g, 9.35 mmol) with 46% yield. Pure **(X1)** was analyzed by LC/MS with **LC/MS method D**.

Fmoc-D-Val-D-Thr($\Psi^{Me,Me}$pro)-OH ***(X2) (Figure S2)***

A solution of Fmoc-D-Val-OH (5 g, 14.7 mmol), and NHS (3.4 g, 29.4 mmol) were dissolved in 40 mL of 80%THF/DMF in an ice bath at 0°C. EDCI (5.62 g, 29.4 mmol) was added, the solution

was warmed to RT and stirred for 3 hours. The reaction was concentrated under reduced pressure and diluted with EtOAc. The organic layer was washed 2x with H_2O, concentrated under reduced pressure, and resuspended in 50 mL of acetone. 8.7 g (73.5 mmol) of H-D-Thr-OH was dissolved in 15 mL of a 10% Na_2CO_3 (w/v) aqueous solution. The D-Thr solution was added to the acetone solution and stirred at RT overnight. The reaction was again concentrated under reduced pressure and diluted with H_2O. It was cooled to 0°C and slowly acidified with 2 N HCl to a pH <3. The dipeptide was extracted with EtOAc and washed with H_2O (3x) and brine (2x). The organic layer was dried with Na_2SO_4 and evaporated under reduced pressure. The crude dipeptide Fmoc-D-Val-D-Thr-OH was lyophilized in 80% ACN/H_2O and used without further purification.

Crude Fmoc-D-Val-D-Thr-OH (6 g, 13.6 mmol), 2,2-DMP (11.3 g, 109 mmol), and PPTS (683 mg, 2.72 mmol) were dissolved in 226 mL of dry THF at RT. The RB flask was fitted with a reflux condenser and placed in an oil bath. The reaction was heated to reflux for 24 hours. The reaction was cooled to RT and the PPTS was neutralized with 500 µL of triethylamine. The THF was removed under reduced pressure and the reaction was diluted with EtOAc. The organic layer was washed 3x with H_2O. The EtOAc layer was dried with Na_2SO_4 and purified by reverse-phase flash chromatography with a H_2O/ACN mixture (10-90% ACN over 35 min) to afford Fmoc-D-Val-D-Thr($\Psi^{Me,Me}$pro)-OH **(X2)** as a white fluffy powder (3.4 g, 7.07 mmol) with 52% yield. Pure **(X2)** was analyzed by LC/MS with **LC/MS method D**.

Fmoc-D-Asp(tBu)-D-Ser($\Psi^{Me,Me}$pro)-OH ***(X3) (Figure S3)***

A solution of Fmoc-D-Asp(tBu)-OH (10 g, 24.3 mmol), and NHS (5.6 g, 48.6 mmol) were dissolved in 67 mL of 80%THF/DMF in an ice bath at 0°C. EDCI (9.3 g, 48.6 mmol) was added, the solution was warmed to RT and stirred for 3 hours. The reaction was concentrated under reduced pressure and diluted with EtOAc. The organic layer was washed 2x with H_2O, concentrated under reduced pressure, and resuspended in 100 mL of acetone. 10.2 g (97.3 mmol) of H-D-Ser-OH was dissolved in 30 mL of a 10% Na_2CO_3 (w/v) aqueous solution. The D-Ser solution was added to the acetone solution and stirred at RT overnight. The reaction was again concentrated under reduced pressure and diluted with H_2O. It was cooled to 0°C and slowly acidified with 2 N HCl to a pH <3. The dipeptide was extracted with EtOAc and washed with H_2O (3x) and brine (2x). The organic layer was dried with Na_2SO_4 and evaporated under reduced pressure. The crude dipeptide Fmoc-D-Asp(tBu)-D-Ser-OH was lyophilized in 75% ACN/H_2O and used without further purification.

Crude Fmoc-D-Asp(tBu)-D-Ser-OH (6 g, 12 mmol), 2,2-DMP (10.03 g, 96.4 mmol), and PPTS (605 mg, 2.41 mmol) were dissolved in 100 mL of dry THF at RT. The RB flask was fitted with a reflux condenser and placed in an oil bath. The reaction was heated to reflux for 24 hours. The reaction was cooled to RT and the PPTS was neutralized with 500 µL of triethylamine. The THF was removed under reduced pressure and the reaction was diluted with EtOAc. The organic layer was washed 3x with H_2O. The EtOAc layer was dried with Na_2SO_4 and purified by reverse-phase flash chromatography with a H_2O/ACN mixture (25-80% ACN over 50 min) to afford Fmoc-D-Asp(tBu)-D-Ser($\Psi^{Me,Me}$pro)-OH **(X3)** as a white fluffy powder (1.3 g, 2.4 mmol) with 20% yield. Pure **(X3)** was analyzed by LC/MS with **LC/MS method D**.

Fmoc-D-Leu-D-Thr($\Psi^{Me,Me}$pro)-OH ***(X4) (Figure S4)***

A solution of Fmoc-D-Leu-OH (5 g, 14.2 mmol), and NHS (3.3 g, 28.4 mmol) were dissolved in 35 mL of 80%THF/DMF in an ice bath at 0°C. EDCI (5.4 g, 28.4 mmol) was added, the solution was warmed to RT and stirred for 3 hours. The reaction was concentrated under reduced pressure and diluted with EtOAc. The organic layer was washed 2x with H_2O, concentrated

under reduced pressure, and resuspended in 50 mL of acetone. 6.7 g (56.8 mmol) of H-D-Thr-OH was dissolved in 15 mL of a 10% Na_2CO_3 (w/v) aqueous solution. The D-Thr solution was added to the acetone solution and stirred at RT overnight. The reaction was again concentrated under reduced pressure and diluted with H_2O. It was cooled to 0°C and slowly acidified with 2 N HCl to a pH <3. The dipeptide was extracted with EtOAc and washed with H_2O (3x) and brine (2x). The organic layer was dried with Na_2SO_4 and evaporated under reduced pressure. The crude dipeptide Fmoc-D-Leu-D-Thr-OH was lyophilized in 90% ACN/H_2O and used without further purification.

Crude Fmoc-D-Leu-D-Thr-OH (3 g, 6.6 mmol), 2,2-DMP (6.9 g, 66.1 mmol), and PPTS (497 mg, 1.98 mmol) were dissolved in 100 mL of dry THF at RT. The RB flask was fitted with a reflux condenser and placed in an oil bath. The reaction was heated to reflux for 24 hours. The reaction was cooled to RT and the PPTS was neutralized with 500 µL of triethylamine. The THF was removed under reduced pressure and the reaction was diluted with EtOAc. The organic layer was washed 3x with H_2O. The EtOAc layer was dried with Na_2SO_4 and purified by reverse-phase flash chromatography with a H_2O/ACN mixture (10-100% ACN over 40 min) to afford Fmoc-D-Leu-D-Thr($^{\Psi Me,Me}$pro)-OH **(X4)** as a white fluffy powder (1.2 g, 2.4 mmol) with 36% yield. Pure **(X4)** was analyzed by LC/MS with **LC/MS method D**.

<u>*Fmoc-D-Lys(Boc)-D-Ser($\Psi^{Me,Me}$pro)-OH* ***(X5) (Figure S5)***</u>

A solution of Fmoc-D-Lys(Boc)-OH (6.6 g, 14.2 mmol), and NHS (3.3 g, 28.4 mmol) were dissolved in 35 mL of 80%THF/DMF in an ice bath at 0°C. EDCI (5.4 g, 28.4 mmol) was added, the solution was warmed to RT and stirred for 4 hours. The reaction was concentrated under reduced pressure and diluted with EtOAc. The organic layer was washed 2x with H_2O, concentrated under reduced pressure, and resuspended in 50 mL of acetone. 5.96 g (56.8 mmol) of H-D-Ser-OH was dissolved in 15 mL of a 10% Na_2CO_3 (w/v) aqueous solution. The D-Ser solution was added to the acetone solution and stirred at RT overnight. The reaction was again concentrated under reduced pressure and diluted with H_2O. It was cooled to 0°C and slowly acidified with 2 N HCl to a pH <3. The dipeptide was extracted with EtOAc and washed with H_2O (3x) and brine (2x). The organic layer was dried with Na_2SO_4 and evaporated under reduced pressure. The crude dipeptide Fmoc-D-Lys(Boc)-D-Ser-OH was lyophilized in 75% ACN/H_2O and used without further purification.

Crude Fmoc-D-Lys(Boc)-D-Ser-OH (3.5 g, 6.3 mmol), 2,2-DMP (6.6 g, 63.1 mmol), and PPTS (402 mg, 1.6 mmol) were dissolved in 100 mL of dry THF at RT. The RB flask was fitted with a reflux condenser and placed in an oil bath. The reaction was heated to reflux for 24 hours. The reaction was cooled to RT and the PPTS was neutralized with 500 µL of triethylamine. The THF was removed under reduced pressure and the reaction was diluted with EtOAc. The organic layer was washed 3x with H_2O. The EtOAc layer was dried with Na_2SO_4 and purified by reverse-phase flash chromatography with a H_2O/ACN mixture (20-90% ACN over 50 min) to afford Fmoc-D-Lys(Boc)-D-Ser($\Psi^{Me,Me}$pro)-OH **(X5)** as a white fluffy powder (1.1 g, 1.8 mmol) with 29% yield. Pure **(X5)** was analyzed by LC/MS with **LC/MS method D**.

<u>*Fmoc-D-Asp(tBu)-D-Thr($\Psi^{Me,Me}$pro)-OH* ***(X6) (Figure S6)***</u>

A solution of Fmoc-D-Asp(tBu)-OH (5 g, 12.2 mmol), and NHS (2.8 g, 24.4 mmol) were dissolved in 32 mL of 80%THF/DMF in an ice bath at 0°C. EDCI (4.6 g, 24.4 mmol) was added, the solution was warmed to RT and stirred for 4 hours. The reaction was concentrated under reduced pressure and diluted with EtOAc. The organic layer was washed 2x with H_2O, concentrated under reduced pressure, and resuspended in 50 mL of acetone. 5.8 g (48.6 mmol) of H-D-Thr-OH was dissolved in 15 mL of a 10% Na_2CO_3 (w/v) aqueous solution. The D-Thr

solution was added to the acetone solution and stirred at RT overnight. The reaction was again concentrated under reduced pressure and diluted with H_2O. It was cooled to 0°C and slowly acidified with 2 N HCl to a pH <3. The dipeptide was extracted with EtOAc and washed with H_2O (3x) and brine (2x). The organic layer was dried with Na_2SO_4 and evaporated under reduced pressure. The crude dipeptide Fmoc-D-Asp(tBu)-D-Thr-OH was lyophilized in 90% ACN/H_2O and used without further purification.

Crude Fmoc-D-Asp(tBu)-D-Thr-OH (4.5 g, 8.8 mmol), 2,2-DMP (9.2 g, 87.9 mmol), and PPTS (882 mg, 3.51 mmol) were dissolved in 100 mL of dry THF at RT. The RB flask was fitted with a reflux condenser and placed in an oil bath. The reaction was heated to reflux for 24 hours. The reaction was cooled to RT and the PPTS was neutralized with 600 µL of triethylamine. The THF was removed under reduced pressure and the reaction was diluted with EtOAc. The organic layer was washed 3x with H_2O. The EtOAc layer was dried with Na_2SO_4 and purified by reverse-phase flash chromatography with a H_2O/ACN mixture (20-100% ACN over 35 min) to afford Fmoc-D-Asp(tBu)-D-Thr($\Psi^{Me,Me}$pro)-OH **(X6)** as a white fluffy powder (2.2 g, 4.0 mmol) with 46% yield. Pure **(X6)** was analyzed by LC/MS with **LC/MS method D**.

S6 Synthesis and Characterization of Fmoc-D-Glu(AlHx)-OH:

Fmoc-D-Glu(AlHx-Dde)-OH was synthesized in a similar fashion as the previously reported L-version.[1] See **figure S7** for the full synthetic scheme and characterization of Fmoc-D-Glu(AlHx-Dde)-OH.

Dde-Ahx-OH ***(X7)***

6-aminohexanoic acid (18.7 g, 142.9 mmol) was suspended in 250 mL of methanol and DIPEA (18.4 g, 142.9 mmol) and stirred at room temperature. 2-Acetyldimedone (Dde-OH) (20 g, 109.9 mmol) was added, and the reaction was warmed to 37ºC and stirred overnight. The methanol was removed under reduced pressure, and the mixture was suspended in water and acidified with HCl (conc.) to pH 3-4. 6-(Dde-amino)hexanoic acid (Dde-Ahx-OH, **(X7)**) was extracted with methylene chloride (5x, 50 mL) and dried with Na_2SO_4. The product was concentrated under reduced pressure to produce a white solid and was used without further purification.

Dde-Ahx-OAll-OH ***(X8)***

15.02 g (50.9 mmol) of **(X7)** was dissolved in 70 mL of methylene chloride, and EDCI (12.7 g, 66.3 mmol) and DMAP (2.5 g, 20.4 mmol) were added. In a separate flask, (*E*)-2-Butene-1,4-diol (8.96 g, 101.8 mmol) was suspended in 100 mL of methylene chloride. The activated **(X7)** solution was slowly added to the diol solution dropwise, and the reaction was stirred at room temperature for 2 hours. After the reaction was complete, the product was concentrated under reduced pressure and directly purified by reverse-phase flash chromatography with a H_2O/ACN mixture (10-80% ACN over 20 min) to afford 9.78 g (26.8 mmol) of the product ((*E*)-4-hydroxybut-2-en-1-yl 6-(Dde)aminohexanoate)(Dde-Ahx-OAll-OH, **(X8)**) in 53% yield. Pure **(X8)** was analyzed by LC/MS with **LC/MS method D**.

Fmoc-D-Glu(AlHx-Dde)-OH ***(X9)***

Fmoc-D-Glu-OtBu (1.4 g, 3.3 mmol) was dissolved in 6 mL of methylene chloride, and EDCI (655 mg, 3.43 mmol) and 133 mg of DMAP (1.1 mmol) were added. 1 g (2.74 mmol) of **(X8)** was added, and the reaction was stirred at room temperature for 3 hours. After the reaction was complete, the solvent was removed under reduced pressure. 10 mL of TFA were then added, and the reaction was stirred for 6 hours. After evaporation of the TFA, the final compound was purified by reverse-phase flash chromatography with a H_2O/ACN mixture (10-95% ACN over 35

min) to afford 1.2 g (1.6 mmol) of Fmoc-D-Glu(AlHx-Dde)-OH **(X9)** in 60% yield. Pure **(X9)** was analyzed by LC/MS with **LC/MS method D**.

S7 Synthesis of L-Streptavidin – Initial Peptide Segments:

L-SA1(AlHx-K_6) **(1)** *(**Figure S8**)*

H--AE(AlHx-K_6)AGITGTWYNQLGSTFIVTAGAD(Tmb)GALTGTYES--$NHNH_2$. Underlined dipeptide sequences indicate pseudoprolines used. (Tmb)G and Boc-L-Ala were also used where underlined. Glu(AlHx) is also underlined. **(1)** was synthesized using standard SPPS and cleavage conditions as outlined in **S2.** The crude and pure peptides were analyzed by RP-HPLC and LC/MS with **analytical method A** and **LC/MS method A**. Crude peptide was purified with **purification method A** (24.9% isolated yield).

L-SA2 **(2)** *(**Figure S9**)*

H--CVGNAESRYVLTGRYDSAPATD(Tmb)GSGTALGWTVAWKNNYRNAHS--$NHNH_2$. Underlined dipeptide sequences indicate pseudoprolines used. (Tmb)G was also used where underlined. **(2)** was synthesized using standard SPPS and cleavage conditions as outlined in **S2.** The crude and pure peptides were analyzed by RP-HPLC and LC/MS with **analytical method A** and **LC/MS method A**. Crude peptide was purified with **purification method B** (29.2% isolated yield).

L-SA3 **(3)** *(**Figure S10**)*

H--CTTWSGQYVG(Tmb)GAEARINTQWLLTSGTTEANAWKSTLVGHDTFTKVKPSAA--S-OH. Underlined dipeptide sequences indicate pseudoprolines used. (Tmb)G was also used where underlined. **(3)** was synthesized using standard SPPS and cleavage conditions as outlined in **S2.** The crude and pure peptides were analyzed by RP-HPLC and LC/MS with **analytical method A** and **LC/MS method A**. Crude peptide was purified with **purification method C** (31.2% isolated yield).

S8 Synthesis of L-Streptavidin – Ligation products and Full-Length Protein:

L-SA1(AlHx-K_6) ***(1)*** *to L-SA2* ***(2)*** *NCL and Desulfurization (**Figure S11, S12**)*

Native chemical ligation between L-SA1(AlHx-K_6) **(1)** and L-SA2 **(2)** was performed using the peptide hydrazide method described in **S4**. 32.2 mg of **(1)** (7.5 µmol) was dissolved in 1.1 mL (7.1 mM) of activation buffer, and the pH was adjusted to 3. The peptide was cooled to -20°C for 10 min, and 225 µL of 500 mM $NaNO_2$ pH 3 was added to 106 mM (15 eq. to **(1)**). The peptide was incubated at -20°C for 20 min. 1,070 µL of 350 mM MPAA, pH 6.5 (355 mM, 50 eq. to **(1)**) was added to the activated peptide solution and the solution was stirred at rt for 1-2 min. 42 mg of **(2)** (9 µmol) was dissolved in 1 mL (8 mM) of ligation buffer and 1000 µL (1.2 eq) of the **(2)** solution was added to the **(1)** solution, which was then pH adjusted to 6.8. After 2 hours, the reaction was transferred to a slide-a-lyzer cassette (3.5K MWCO) and dialyzed against 200 mL of fresh ligation buffer with 5 mM TCEP at rt for 3 hours. The cassette was transferred to a new beaker with 200 mL of fresh ligation buffer with 5 mM TCEP and again dialyzed overnight at 4 °C.

Next, desulfurization was performed as described in **S4**. 4 mL of 400 mM reduced glutathione, 200 mM VA-044 in desulfurization buffer was prepared, along with a solution of 600 mM TCEP in desulfurization buffer. 4 mL of the L-SA1-2(AlHx-K_6)_C34 **(4)** were mixed with equal volumes

of the VA-044/GSH solution and TCEP solution were mixed and carefully pH adjusted to 6.5. The final concentrations were 67 mM VA-044, 133 mM GSH, 200 mM TCEP, and 0.63 mM **(4)**. The reaction was then covered with argon gas and mixed in an oil bath at 50°C for 4 hours. Once the desulfurization had stalled (monitored by LC/MS), the reaction was diluted 1:1 with 0.1% TFA in water to lower the 6 M GdmHCl to 3 M. After spinning the sample at 14,000 rpm for 15 min, the supernatant was filtered and purified by RP-HPLC with **purification method D** to yield 25.2 mg of L-SA1-2(AlHx-K_6) **(5)** (38% yield). The ligation reaction was analyzed by RP-HPLC and LC/MS with **analytical method D** and **LC/MS method B** . Pure **(5)** was analyzed using **analytical method B** and **LC/MS method A**. The lyophilized powder was then stored until the next reaction.

L-SA1-2(AlHx-K6) ***(5)*** *to L-SA3* ***(3)*** *NCL and Desulfurization (****Figure S13, S14****)*

Native chemical ligation between L-SA1-2(AlHx-K_6) **(5)** and L-SA3 **(3)** was performed using the peptide hydrazide method described in **S4**. 18.1 mg of **(5)** (2.04 µmol) was dissolved in 0.41 mL (4.3 mM) of activation buffer, and the pH was adjusted to 3. The peptide was cooled to -20°C for 10 min, and 40.8 µL of 500 mM $NaNO_2$ pH 3 was added to 43 mM (10 eq. to **(5)**). The peptide was incubated at -20°C for 20 min. 291 µL of 350 mM MPAA, pH 6.5 (215 mM, 50 eq. to **(5)**) was added to the activated peptide solution and the solution was stirred at rt for 1-2 min. 13.6 mg of **(3)** (2.5 µmol) was dissolved in 335 µL (7.6 mM) of ligation buffer and 317 µL (1.15 eq) of the **(3)** solution was added to the **(5)** solution, which was then pH adjusted to 6.8. After 2 hours, the reaction was transferred to a slide-a-lyzer cassette (3.5K MWCO) and dialyzed against 200 mL of fresh ligation buffer with 5 mM TCEP at rt for 3 hours. The cassette was transferred to a new beaker with 200 mL of fresh ligation buffer with 5 mM TCEP and again dialyzed overnight at 4°C.

Next, desulfurization was performed as described in **S4**. 3 mL of 400 mM reduced glutathione, 200 mM VA-044 in desulfurization buffer was prepared, along with a solution of 600 mM TCEP in desulfurization buffer. 1.5 mL of the L-SA1-3(AlHx-K_6)_C77 **(6)** were mixed with equal volumes of the VA-044/GSH solution and TCEP solution were mixed and carefully pH adjusted to 6.6. The final concentrations were 67 mM VA-044, 133 mM GSH, 200 mM TCEP, and 0.41 mM **(6)**. The reaction was then covered with argon gas and mixed in an oil bath at 50°C for 1.5 hours. Once the desulfurization had finished (monitored by LC/MS), the reaction was diluted 1:1 with 0.1% TFA in water to lower the 6 M GdmHCl to 3 M. After spinning the sample at 14,000 rpm for 15 min, the supernatant was filtered and purified by RP-HPLC with **purification method E** to yield 12 mg of L-SA1-3(AlHx-K_6) **(7)** (41% yield). The ligation reaction was analyzed by LC/MS with **LC/MS method C**. Pure **(7)** was analyzed using **analytical method C** and **LC/MS method A**. The lyophilized powder was then stored until the next reaction.

Removal of AlHx-K_6 From L-SA1-3(AlHx-K_6) ***(7)*** *and Dialysis (****Figure S15****)*

AlHx-K_6 removal was performed as described in **S4**. 5.7 mg of L-SA1-3(AlHx-K_6) **(7)** was dissolved in 350 µL of degassed ligation buffer (1.14 mM). 1 mL of 188 mM $Pd(OAc)_2$ in DMF, 1 mL of 350 mM reduced glutathione in ligation buffer, and 0.1 mL of 850 mM TPPTS in H_2O were prepared in degassed solvents and buffers. 100 µL of the $Pd(OAc)_2$ and TPPTS solutions were mixed and vortex for 30 s (this is now 100 mM $Pd(TPPTS)_4$). 70 µL of the $Pd(TPPTS)_4$ solution (20 mM) and 10 µL of the glutathione solution (10 mM) were added to **(7)** and the pH was carefully adjusted to 8. The reaction was mixed on a rotisserie at 37°C for 1.5 hours. After the reaction was finished (monitored by LC/MS) an equal volume of 1M DTT in H_2O was added to the reaction to quench the Pd. After 30 min, the reaction was transferred to a slide-a-lyzer cassette (7K MWCO) and dialyzed against 200 mL of fresh ligation buffer with 100 mM DTT

overnight at 4°C. The cassette was transferred to a new beaker with 200 mL of fresh ligation buffer with 100 mM DTT and again dialyzed overnight at 4°C. Finally, the cassette was transferred to a new beaker with 200 mL of 80 mM Tris, 6 M GdmHCl, pH 8 and dialyzed overnight at 4°C. Pure L-SA1-3 **(8)** was analyzed using **analytical method C** and **LC/MS method A**. **(8)** (now L-SA) was removed from the cassette, flash frozen, and stored at -80°C until folding (described below).

S9 Synthesis of D-Streptavidin – Initial Peptide Segments

D-SA1(AlHx-K_6) **(1*)** *(**Figure S16**)*

H--AE(AlHx-K_6)AGITGTWYNQLGSTFIVTAGAD(Tmb)GALTGTYES--$NHNH_2$. Underlined dipeptide sequences indicate pseudoprolines used. (Tmb)G and Boc-D-Ala were also used where underlined. Glu(AlHx) is also underlined. **(1*)** was synthesized using standard SPPS and cleavage conditions as outlined in **S2.** The crude and pure peptides were analyzed by RP-HPLC and LC/MS with **analytical method A** and **LC/MS method A**. Crude peptide was purified with **purification method A** (18.5% isolated yield).

D-SA2 **(2*)** *(**Figure S17**)*

H--CVGNAESRYVLTGRYDSAPATD(Tmb)GSGTALGWTVAWKNNYRNAHS--$NHNH_2$. Underlined dipeptide sequences indicate pseudoprolines used. (Tmb)G was also used where underlined. **(2*)** was synthesized using standard SPPS and cleavage conditions as outlined in **S2.** The crude and pure peptides were analyzed by RP-HPLC and LC/MS with **analytical method A** and **LC/MS method A**. Crude peptide was purified with **purification method B** (19.1% isolated yield).

D-SA3 **(3*)** *(**Figure S18**)*

H--CTTWSGQYVG(Tmb)GAEARINTQWLLTSGTTEANAWKSTLVGHDTFTKVKPSAA--S-OH. Underlined dipeptide sequences indicate pseudoprolines used. (Tmb)G was also used where underlined. **(3*)** was synthesized using standard SPPS and cleavage conditions as outlined in **S2.** The crude and pure peptides were analyzed by RP-HPLC and LC/MS with **analytical method A** and **LC/MS method A**. Crude peptide was purified with **purification method C** (19.0% isolated yield).

S10 Synthesis of D-Streptavidin – Ligation products and Full-Length Protein

D-SA1(AlHx-k_6) ***(1*)*** *to D-SA2* ***(2*)*** *NCL and Desulfurization (**Figure S19,S20**)*

Native chemical ligation between D-SA1(AlHx-k_6) **(1*)** and D-SA2 **(2*)** was performed using the peptide hydrazide method described in **S4**. 39.8 mg of **(1*)** (9.3 µmol) was dissolved in 1.2 mL (7.8 mM) of activation buffer, and the pH was adjusted to 3. The peptide was cooled to -20°C for 10 min, and 278 µL of 500 mM $NaNO_2$ pH 3 was added to 117 mM (15 eq. to **(1*)**). The peptide was incubated at -20°C for 20 min. 1.3 mL of 350 mM MPAA, pH 6.5 (390 mM, 50 eq. to **(1*)**) was added to the activated peptide solution and the solution was stirred at rt for 1-2 min. 52 mg of **(2*)** (11.1 µmol) was dissolved in 1.4 mL (8 mM) of ligation buffer and 1.4 mL (1.2 eq) of the **(2*)** solution was added to the **(1*)** solution, which was then pH adjusted to 6.9. After 2 hours, the reaction was transferred to a slide-a-lyzer cassette (3.5K MWCO) and dialyzed against 200 mL of fresh ligation buffer with 5 mM TCEP at rt for 3 hours. The cassette was

transferred to a new beaker with 200 mL of fresh ligation buffer with 5 mM TCEP and again dialyzed overnight at 4°C.

Next, desulfurization was performed as described in **S4**. 6 mL of 400 mM reduced glutathione, 200 mM VA-044 in desulfurization buffer was prepared, along with a solution of 600 mM TCEP in desulfurization buffer. 4.5 mL of the D-SA1-2(AlHx-k_6)_c34 **(4*)** were mixed with equal volumes of the VA-044/GSH solution and TCEP solution were mixed and carefully pH adjusted to 6.6 The final concentrations were 67 mM VA-044, 133 mM GSH, 200 mM TCEP, and 0.58 mM **(4*)**. The reaction was then covered with argon gas and mixed in an oil bath at 50°C for 4 hours. Once the desulfurization had stalled (monitored by LC/MS), the reaction was diluted 1:1 with 0.1% TFA in water to lower the 6 M GdmHCl to 3 M. After spinning the sample at 14,000 rpm for 15 min, the supernatant was filtered and purified by RP-HPLC with **purification method D** to yield 32 mg of D-SA1-2(AlHx-k_6) **(5*)** (39% yield). The ligation reaction was analyzed by RP-HPLC and LC/MS with **analytical method D** and **LC/MS method B**. Pure **(5*)** was analyzed using **analytical method B** and **LC/MS method A**. The lyophilized powder was then stored until the next reaction.

D-SA1-2(AlHx-k_6) ***(5*)*** *to D-SA3* ***(3*)*** *NCL and Desulfurization (**Figure S21,S22**):*

Native chemical ligation between desulfurized D-SA1-2(AlHx-k_6) **(5*)** and D-SA3 **(3*)** was performed using the peptide hydrazide method described in **S4**. 17.2 mg of **(5*)** (1.95 µmol) was dissolved in 0.54 mL (3.6 mM) of activation buffer, and the pH was adjusted to 3. The peptide was cooled to -20°C for 10 min, and 39 µL of 500 mM $NaNO_2$ pH 3 was added to 36 mM (10 eq. to **(5*)**). The peptide was incubated at -20°C for 20 min. 278 µL of 350 mM MPAA, pH 6.5 (180 mM, 50 eq. to **(5*)**) was added to the activated peptide solution and the solution was stirred at rt for 1-2 min. 12.2 mg of **(3*)** (2.2 µmol) was dissolved in 320 µL (7 mM) of ligation buffer and 320 µL (1.15 eq) of the **(3*)** solution was added to the **(5*)** solution, which was then pH adjusted to 6.9. After 2 hours, the reaction was transferred to a slide-a-lyzer cassette (3.5K MWCO) and dialyzed against 200 mL of fresh ligation buffer with 5 mM TCEP at rt for 3 hours. The cassette was transferred to a new beaker with 200 mL of fresh ligation buffer with 5 mM TCEP and again dialyzed overnight at 4°C.

Next, desulfurization was performed as described in **S4**. 3 mL of 400 mM reduced glutathione, 200 mM VA-044 in desulfurization buffer was prepared, along with a solution of 600 mM TCEP in desulfurization buffer. 1.5 mL of the D-SA1-3(AlHx-k_6)_c77 **(6*)** were mixed with equal volumes of the VA-044/GSH solution and TCEP solution were mixed and carefully pH adjusted to 6.6. The final concentrations were 67 mM VA-044, 133 mM GSH, 200 mM TCEP, and 0.48 mM **(6*)**. The reaction was then covered with argon gas and mixed in an oil bath at 50°C for 1.5 hours. Once the desulfurization had finished (monitored by LC/MS), the reaction was diluted 1:1 with 0.1% TFA in water to lower the 6 M GdmHCl to 3 M. After spinning the sample at 14,000 rpm for 15 min, the supernatant was filtered and purified by RP-HPLC with **purification method E** to yield 7 mg of D-SA1-3(AlHx-k_6) **(7*)** (25% yield). The ligation reaction was analyzed by LC/MS with **LC/MS method C** . Pure **(7*)** was analyzed using **analytical method C** and **LC/MS method A**. The lyophilized powder was then stored until the next reaction.

Removal of AlHx-k_6 From D-SA1-3(AlHx-k_6) ***(7*)*** *and Dialysis (**Figure S23**)*

AlHx-k_6 removal was performed as described in **S4**. 7 mg of D-SA1-3(AlHx-k_6) **(7*)** was dissolved in 175 µL of degassed ligation buffer (2.7 mM). 1 mL of 188 mM $Pd(OAc)_2$ in DMF, 1 mL of 350 mM reduced glutathione in ligation buffer, and 0.1 mL of 850 mM TPPTS in H_2O were prepared in degassed solvents and buffers. 100 µL of the $Pd(OAc)_2$ and TPPTS solutions were

mixed and vortex for 30 s (this is now 100 mM Pd(TPPTS)$_4$). 38 µL of the Pd(TPPTS)$_4$ solution (20 mM) and 6 µL of the glutathione solution (10 mM) were added to **(7*)** and the pH was carefully adjusted to 8. The reaction was mixed on a rotisserie at 37°C for 1.5 hours. After the reaction was finished (monitored by LC/MS) an equal volume of 1M DTT in H_2O was added to the reaction to quench the Pd. After 30 min, the reaction was transferred to a slide-a-lyzer cassette (7K MWCO) and dialyzed against 200 mL of fresh ligation buffer with 100 mM DTT overnight at 4°C. The cassette was transferred to a new beaker with 200 mL of fresh ligation buffer with 100 mM DTT and again dialyzed overnight at 4°C. Finally, the cassette was transferred to a new beaker with 200 mL of 80 mM Tris, 6 M GdmHCl, pH 8 and dialyzed overnight at 4°C. Pure D-SA1-3 **(8*)** was analyzed using **analytical method C** and **LC/MS method A**. **(8*)** (now D-SA) was removed from the cassette, flash frozen, and stored at -80°C until folding (described below).

S11 Folding of Synthetic (L)- and (D)-Streptavidin and Circular Dichroism Analysis

Before using the synthetic material, folding of commercially-available recombinant SA was assessed using a HABA-mediated method. Recombinant SA was reconstituted to a concentration of 60 µM in 80 mM TrisHCl, 6 M GdmHCl, pH 8.0 (Tris buffer). A 300 µL aliquot of this solution was heated at 85°C for 45 minutes. Initial trials confirmed protein unfolding via circular dichroism (CD). Following heating, the sample was centrifuged at 14,000 × g for 5 minutes. The supernatant was collected and diluted 6-fold via dropwise addition into 1.5 mL of 750 µM HABA in 80 mM TrisHCl, pH 8.0, resulting in a total volume of 1.8 mL. This mixture was agitated on an orbital shaker at room temperature for 30 minutes. After incubation, the solution was transferred into a pre-wetted 3.5 kDa MWCO dialysis cassette and dialyzed against 1 L of 80 mM TrisHCl, pH 8.0 with gentle stirring at 4°C. After 8 hours, the buffer was replaced with fresh buffer and dialysis continued overnight. The dialyzed SA was removed from the cassette and centrifuged at 14,000× g for 5 minutes. The resulting supernatant was transferred to a pre-rinsed 10 kDa MWCO spin concentrator and concentrated to approximately 200 µL. To maximize recovery, 100 µL of flowthrough was used to rinse the spin filter, yielding a total recovered volume of 300 µL. NanoDrop A_{280} analysis of the flowthrough confirmed no detectable SA leakage through the spin filter into the flowthrough. The concentrated sample was centrifuged again at 14,000 × g for 5 minutes prior to triplicate A_{280} yield measurements, using 80 mM TrisHCl, pH 8.0 as a blank.This procedure was replicated for synthetic L- and D-streptavidin without the initial heat denaturation step.

For comparison, an alternative dilution-based method reported in previous literature[5] was tested using recombinant SA. The protein was reconstituted as before (60 µM in Tris buffer), and a 300 µL sample was heated at 85°C for 45 minutes. The sample was centrifuged at 14,000 × g for 5 minutes, and the denatured protein was diluted 6-fold into 1.5 mL of 80 mM TrisHCl, pH 8.0, followed by 30 minutes of incubation at room temperature. The sample was then centrifuged again at 14,000 × g for 5 minutes. A pre-rinsed 10 kDa MWCO spin concentrator was used to concentrate the SA back to 300 µL. The protein was further diluted 100-fold in 80 mM TrisHCl, pH 8.0 to a volume of 30 mL and then re-concentrated to 300 µL. The sample was incubated at 4°C overnight and centrifuged before triplicate A_{280} yield measurements.

CD spectra for refolded recombinant SA and synthetic L-SA and D-SA were obtained using a JASCO J-810 circular dichroism spectrometer. Samples and blank 80 mM TrisHCl, pH 8.0 were loaded into a 1 mm quartz cuvette and analyzed at 25°C. Wavelength scans were performed from 280 to 190 nm with a resolution of 1 nm and 1-second averaging time, in triplicate. The

resulting spectra were averaged, blank-subtracted, and normalized to mean residue ellipticity using the formula: [θ] = 100 × θ / (C × l × n), where C is the protein concentration in mM, l is the path length in cm, and n is the number of peptide bonds in the monomer.

S12 Characterization of Synthetic (L)- and (D)-Streptavidin-biotin binding via ITC

ITC Data were collected on a Malvern Panalytical MicroCal iTC$_{200}$. Each run consisted of 20 injections of the ligand into a solution of recombinant, L-, or D-SA. The reference temperature was set to 25˚C with a reference power of 5 µcal/s and initial delay of 60 s. The buffer for all experiments was 50 mM phos, 150 NaCl, pH 7.5. The concentration of the protein and ligand for each experiment are as follows:

1) 27 µM recombinant-SA and 250 µM (+)-biotin
2) 100 µM recombinant-SA and 650 µM (-)-biotin
3) 25 µM synthetic L-SA and 250 µM (+)-biotin
4) 100 µM synthetic L-SA and 650 µM (-)-biotin
5) 100 µM synthetic D-SA and 650 µM (+)-biotin
6) 26 µM synthetic D-SA and 250 µM (-)-biotin

The first ligand injection of each experiment was 0.4 µL and was allowed to equilibrate for 180 s. The remaining 19 injections were 2 µL, also equilibrated for 180 s.

S13 Analysis of Recombinant Streptavidin-biotin interaction through X-ray crystallography

Commercially-available lyophilized recombinant L-SA protein was dissolved in buffer containing Tris-HCl (10 mM) pH 7.0 to final a concentration of 10 mg/ml (0.753 mM). D-Biotin was dissolved in buffer containing Tris-HCl (10 mM) pH 8.0 with gentle heating to a final concentration of 30 mM. L-Biotin was dissolved in PBS buffer to a final concentration of 20 mM. The D-biotin/L-SA and L-biotin/L-SA complexes were formed by mixing 5 µl D-biotin solution and 5 µl L-biotin solution, respectively, with 50 µl L-SA to create mixtures at a final concentration of ~9.1 mg/ml L-SA with 4-fold molar excess of D-biotin to L-SA and 2.6-fold molar excess L-biotin to L-SA. These solutions were incubated overnight at 4˚C and filtered through a 0.2 µm filter prior to use in crystallization trials. Crystals were obtained by sitting drop vapor diffusion by mixing equal amounts (1 µl) biotin-protein complex and screen condition and sealed in chambers where the reservoir contained 500 µL of screen solution. Crystallization conditions were mixed from stock solutions of ammonium sulfate (4.1 M), NaCl (5 M), and Na-acetate buffer (2 M). Final crystallization conditions contained 0.2 M NaCl, 37% ammonium sulfate and 0.1 M Na-acetate buffer pH 4.6. Crystallization trials were incubated at 21°C and crystals suitable for X-ray diffraction were obtained after 10-14 days.

In preparation for data collection, crystals were harvested using a small rayon loop attached to a mounting pin. Crystals were transferred briefly (10 s) to a solution containing mother liquor with 30% added glycerol. Crystals were then quickly plunged into a reservoir of liquid nitrogen and loaded in a cryo-cassette for shipment to the synchrotron. Diffraction data were collected using radiation at 0.96 Å wavelength, with a Pilatus-6M detector on beamline 9-2 at the Stanford Synchrotron Radiation Lightsource (SSRL, National Accelerator Laboratory, Menlo Park, CA). SSRL resources, including Remote Access, Blu-Ice, the Automated Sample Mounting System, and the AUTOXDS script were used for data collection and analysis.[6-9] Diffraction data were processed, integrated, and scaled with XDS[10] and AIMLESS.[11] The D-biotin/L-SA and L-

biotin/L-SA complex datasets were integrated and scaled to 0.95 and 0.94 Å resolution, respectively.

The initial structure of the D-biotin/L-SA complex was determined by molecular replacement (MR), using data limited to 1.7 Å resolution with Phaser in the PHENIX software package.[12] The model (pdb 3RY2) was modified for use as a search model consisting of a dimer of the 3RY2 protein model (streptavidin) without ligand or solvent molecules and where residues W75, W92, and W108 in each of the two monomers were truncated to alanine to act as positive control markers in electron density maps. The result of the MR search (TFZ = 167) recapitulated the crystal packing of the published crystal structure, 3RY2, with a single copy of the streptavidin dimer in the asymmetric unit as expected. Examination of strong features in the resulting electron density maps allowed correction of the intentionally mutated residues (75, 92, and 108), validating the solution. Clear difference density permitted placement of ligand. The process of MR at 1.7 Å resolution was repeated for determination of the initial structure of the L-biotin/L-SA complex. The MR search results (TFZ = 69) and clear features in the electron density maps again confirmed the correctness of the initial solution.

Both structures were rebuilt (COOT)[13] and refined[14, 15] independently through an iterative process of model building and refinement, including the addition of ligand and solvent molecules. Numerous amino acid side chains were modeled in two alternate conformations, to account for detailed features in electron density maps. These features are consistent with alternate side chain conformations seen in 3RY2, and indicate flexibility of these residues. Unbiased electron density guided the placement of L-biotin in two conformations. Data scaling and model refinement statistics can be seen in Table X. Refinement with Refmac5[15] provided a useful comparison of reported values for overall coordinate error (see Table S1). Models were compared by overlapping the core regions of the D-biotin and L-biotin complexes and 3RY2. Residues were chosen from a set of 62 core residues identified by Le Trong et al. We eliminated 9 of these residues which contact the ligand, leaving us with 53 core residues. All overlaps were made using the tetramer, overlapping 212 C-alpha coordinates (4 x 53 core residues). Atomic overlaps were conducted using the program lsqkab[16] in the CCP4[17] suite of programs.

S14 Supplementary Figures, Schemes, and Tables

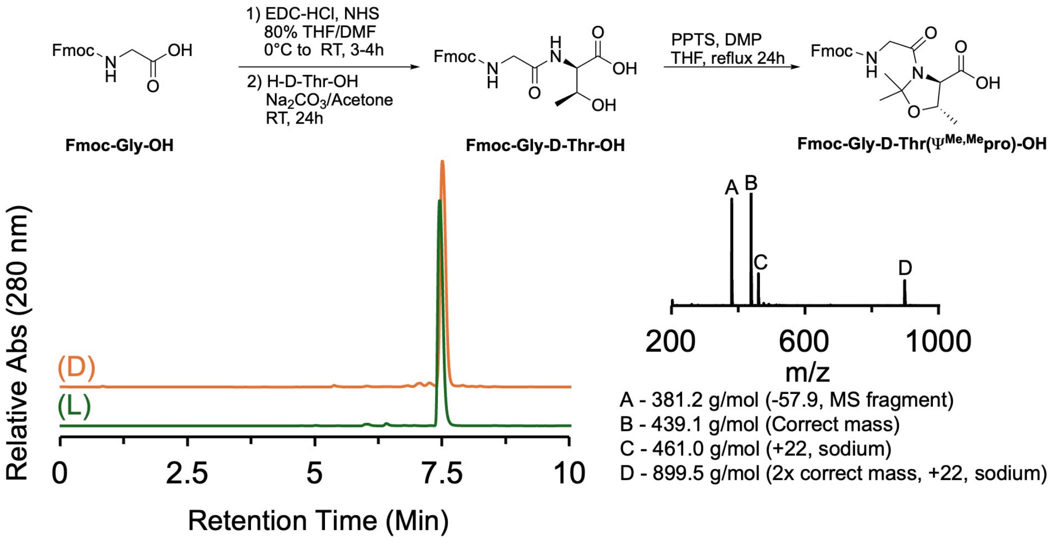

Figure S1. Synthetic scheme and characterization of Fmoc-Gly-D-Thr($\Psi^{Me,Me}$pro)-OH **(X1)**. The LC spectrum of the commercially available L-version is shown for comparison. MS of the pure D-version is also shown. Ion peak A is believed to be MS-induced McLafferty rearrangement resulting in a decrease of 57.9 g/mol, which is present in both L- and D- versions of the pseudoproline dipeptide.

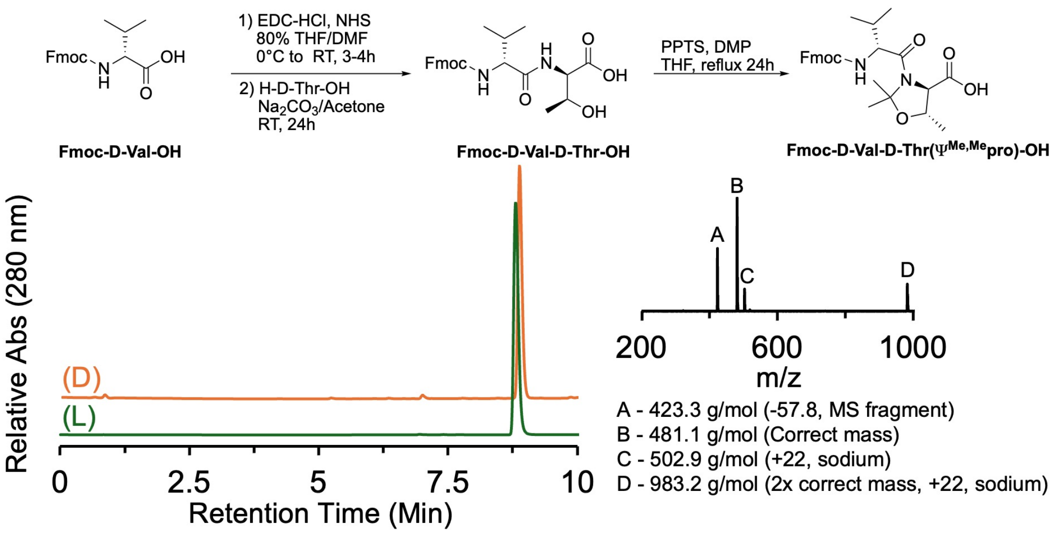

Figure S2. Synthetic scheme and characterization of Fmoc-D-Val-D-Thr($\Psi^{Me,Me}$pro)-OH **(X2)**. The LC spectrum of the commercially available L-version is shown for comparison. MS of the pure D-version is also shown. Ion peak A is believed to be MS-induced McLafferty rearrangement resulting in a decrease of 57.8 g/mol, which is present in both L- and D- versions of the pseudoproline dipeptide.

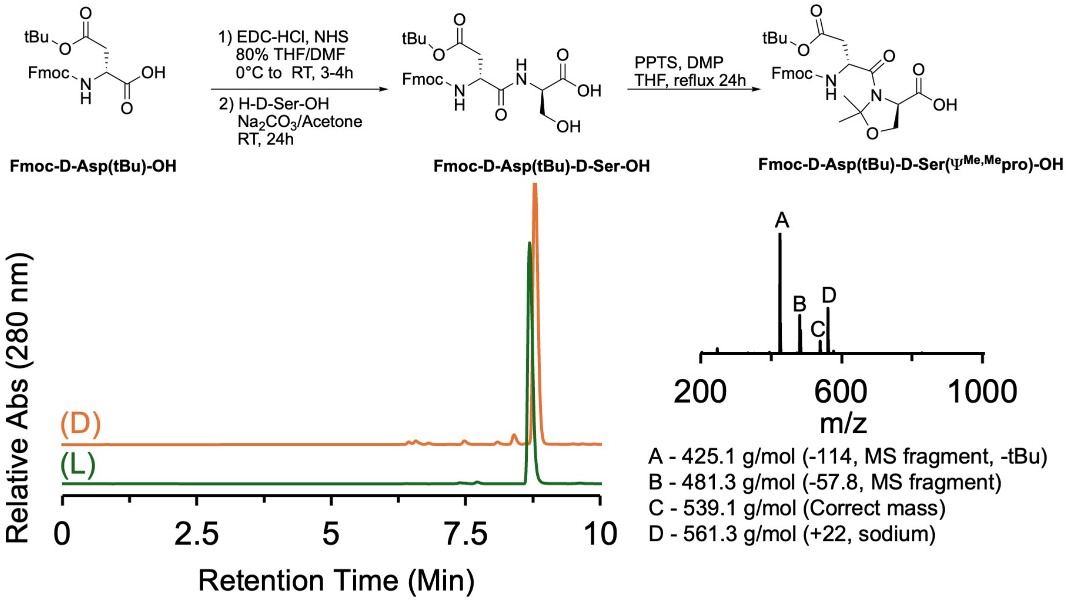

Figure S3. Synthetic scheme and characterization of Fmoc-D-Asp(tBu)-D-Ser($\Psi^{Me,Me}$pro)-OH **(X3)**. The LC spectrum of the commercially available L-version is shown for comparison. MS of the pure D-version is also shown. Ion peak A and B are believed to be MS-induced McLafferty rearrangement fragments with and without the tBu protecting group of the Asp side chain. These fragments show a decrease of 114 and 57.8 g/mol, which is present in both L- and D- versions of the pseudoproline dipeptide.

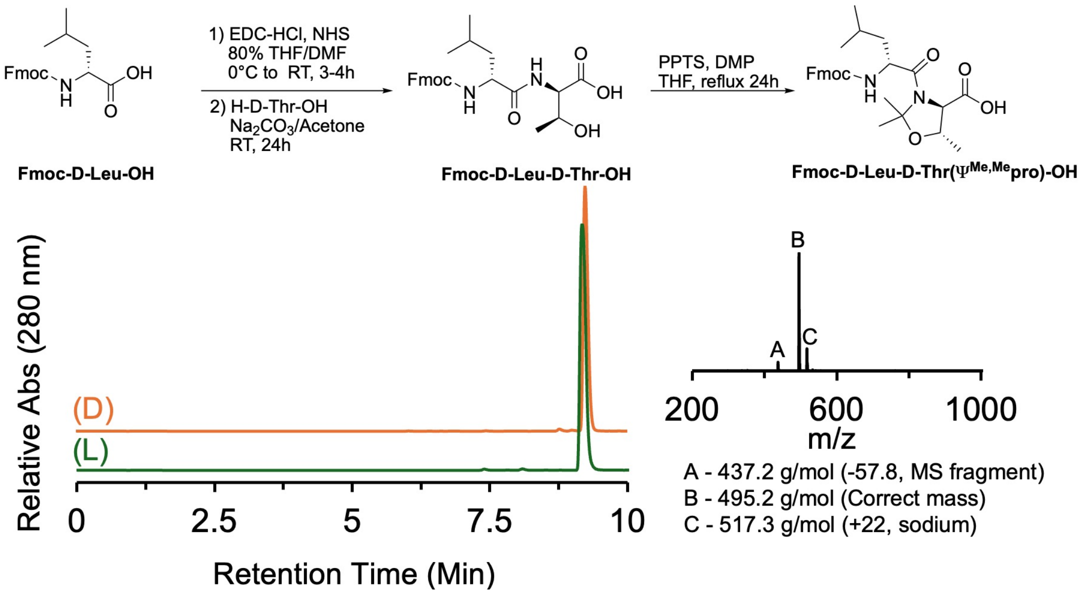

Figure S4. Synthetic scheme and characterization of Fmoc-D-Leu-D-Thr($\Psi^{Me,Me}$pro)-OH **(X4)**. The LC spectrum of the commercially available L-version is shown for comparison. MS of the pure D-version is also shown. Ion peak A is believed to be MS-induced McLafferty rearrangement resulting in a decrease of 57.8 g/mol, which is present in both L- and D- versions of the pseudoproline dipeptide.

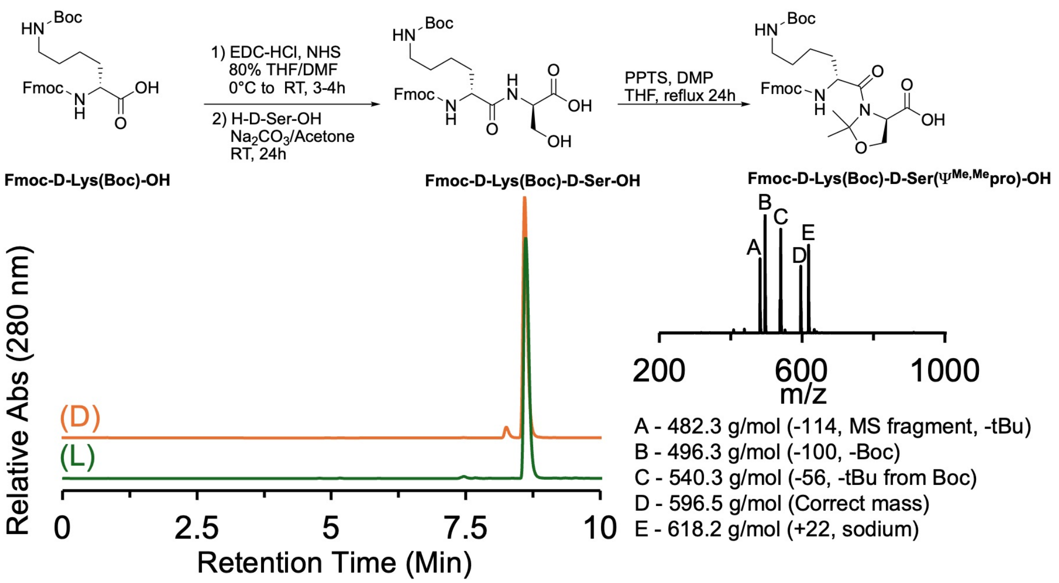

Figure S5. Synthetic scheme and characterization of Fmoc-D-Lys(Boc)-D-Ser($\Psi^{Me,Me}$pro)-OH **(X5)**. The LC spectrum of the commercially available L-version is shown for comparison. MS of the pure D-version is also shown. Several fragment ions are shown which represent different deprotected species and the McLafferty rearrangement fragment. This pattern was also observed in the L-version.

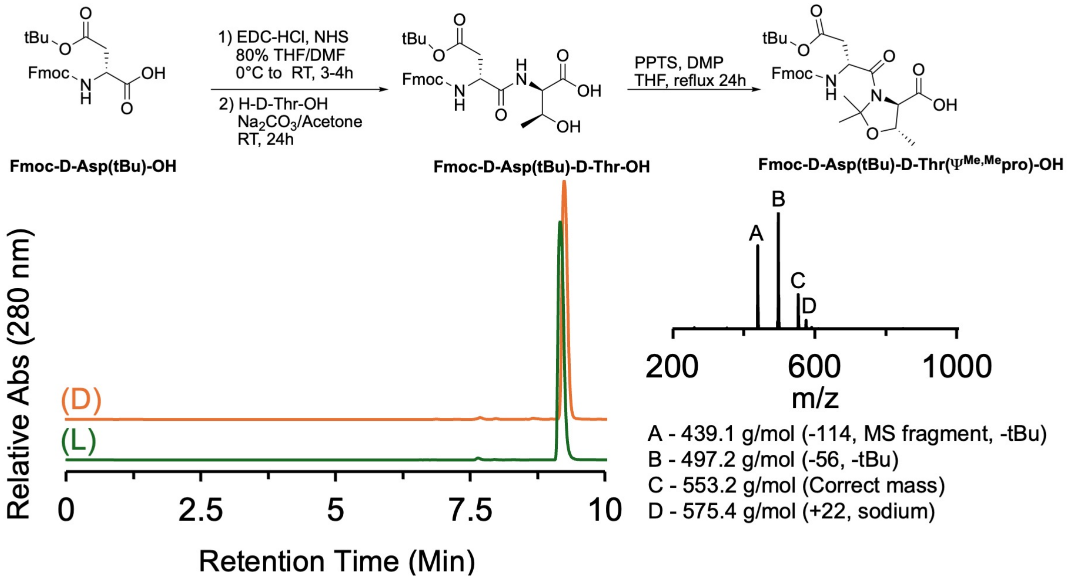

Figure S6. Synthetic scheme and characterization of Fmoc-D-Asp(tBu)-D-Thr($\Psi^{Me,Me}$pro)-OH **(X6)**. The LC spectrum of the commercially available L-version is shown for comparison. MS of the pure D-version is also shown. Ion peak A is believed to be MS-induced McLafferty rearrangement fragments and tBu deprotection of the Asp side chain. This fragment shows a decrease of 114 g/mol, which is present in both L- and D- versions of the pseudoproline dipeptide.

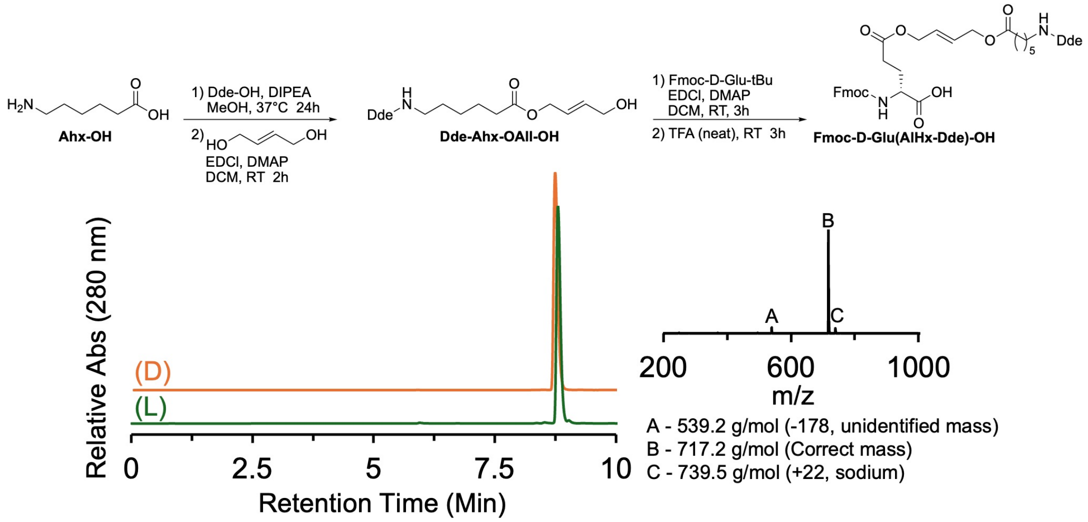

Figure S7. Synthetic scheme and characterization of Fmoc-D-Glu(AlHx-Dde)-OH **(X9)**. LC spectra of both L- and D- versions of the final amino acid are shown for comparison, as well as the MS spectrum of the Fmoc-D-Glu(Alhx-Dde)-OH. An unidentified mass ion of 539.2 g/mol was present in both L- and D- samples.

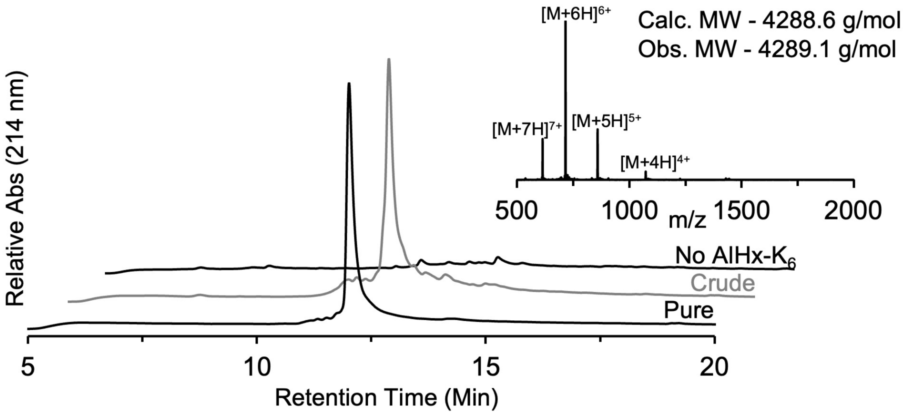

Figure S8. Characterization of L-SA1(AlHx-K_6) **(1)**. This peptide was purified by purification method A with an isolated yield of 24.9% and analyzed by RP-HPLC with analytical method A. MS of the pure peptide is reported. The no AlHx-K_6 chromatogram is L-SA1 without the solubilizing tag. See S7 for more detail.

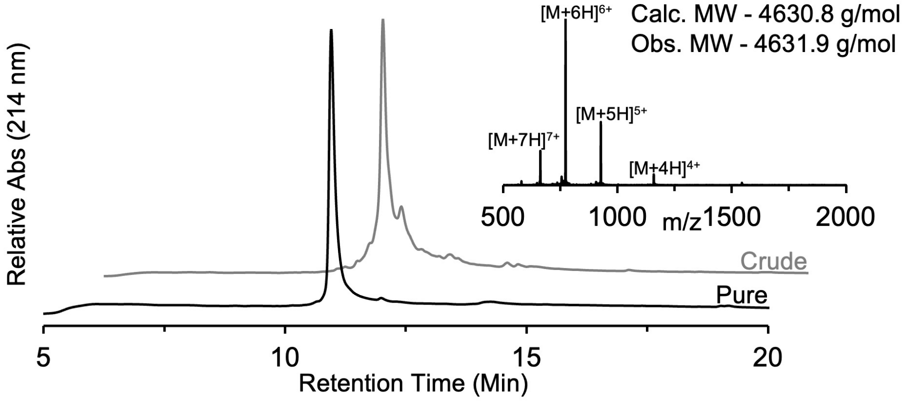

Figure S9. Characterization of L-SA2 **(2)**. This peptide was purified by purification method B with an isolated yield of 29.2% and analyzed by RP-HPLC with analytical method A. MS of the pure peptide is reported. See S7 for more detail.

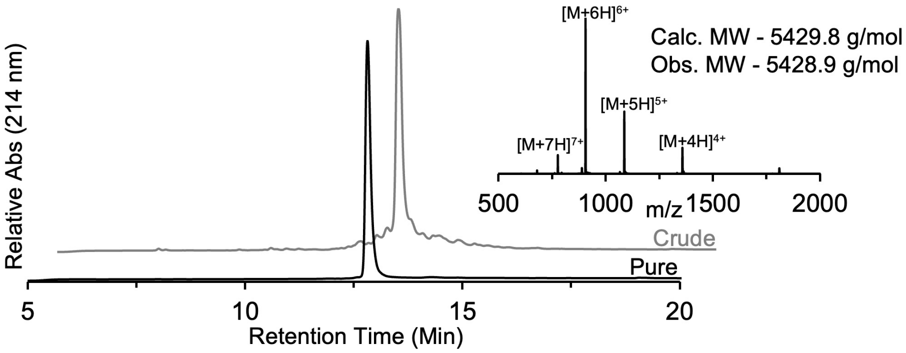

Figure S10. Characterization of L-SA3 **(3)**. This peptide was purified by purification method C with an isolated yield of 31.2% and analyzed by RP-HPLC with analytical method A. MS of the pure peptide is reported. See S7 for more detail.

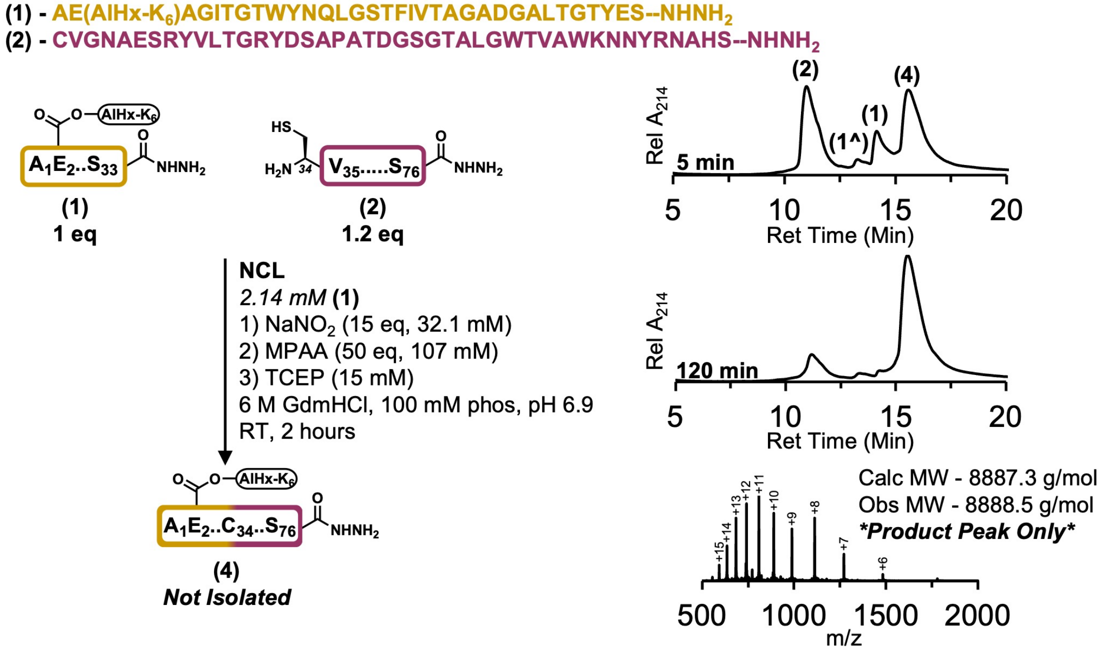

Figure S11. NCL of L-SA1(AlHx-K6) **(1)** and L-SA2 **(2)**. This reaction was monitored by RP-HPLC with analytical method D. In the 5 min timepoint, both the active and hydrolyzed thioester of **(1)** can be observed (**(1^)** denotes hydrolyzed peptide). After 120 min, no active thioester was present and the reaction was stopped. The MS shown is taken only from the TIC peak corresponding to the ligated product L-SA1-2(AlHx-K6)_C34 **(4)** to identify the ligated mass. This product was not purified and carried to the following desulfurization. See S8 for more detail.

(4) - AE(AlHx-K_6)AGITGTWYNQLGSTFIVTAGADGALTGTYES<u>C</u>VGNAESRYVLTGRY DSAPATDGSGTALGWTVAWKNNYRNAHS--$NHNH_2$

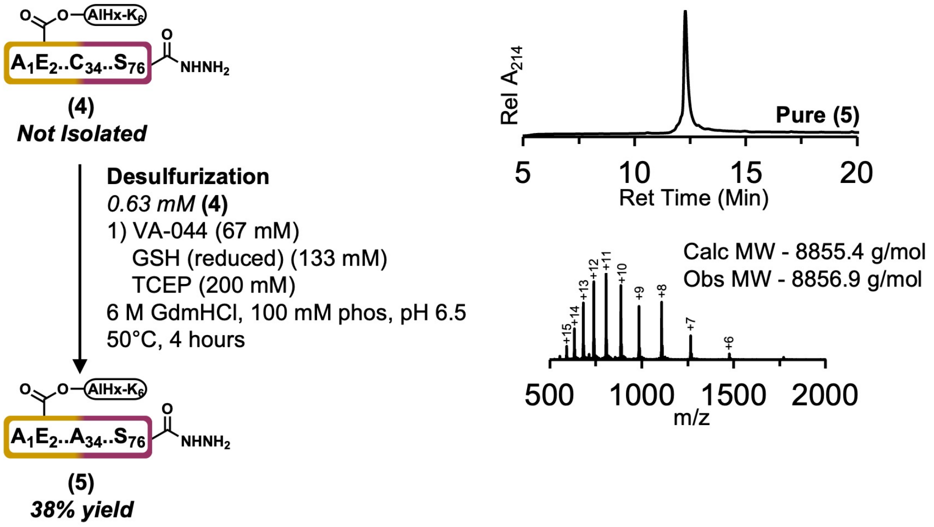

Figure S12. Desulfurization of L-SA1-2(AlHx-K_6)_C34 **(4)**. This reaction was monitored LC/MS with LC/MS method B. After 4 hours, no additional desulfurization was observed, and the ligated product was purified. Pure L-SA1-2(AlHx-K_6) **(5)** RP-HPLC and MS traces are shown. See S8 for more detail.

(5) - AE(AlHx-K$_6$)AGITGTWYNQLGSTFIVTAGADGALTGTYESAVGNAESRYVLTGRY DSAPATDGSGTALGWTVAWKNNYRNAHS--NHNH$_2$
(3) - CTTWSGQYVGGAEARINTQWLLTSGTTEANAWKSTLVGHDTFTKVKPSAAS--OH

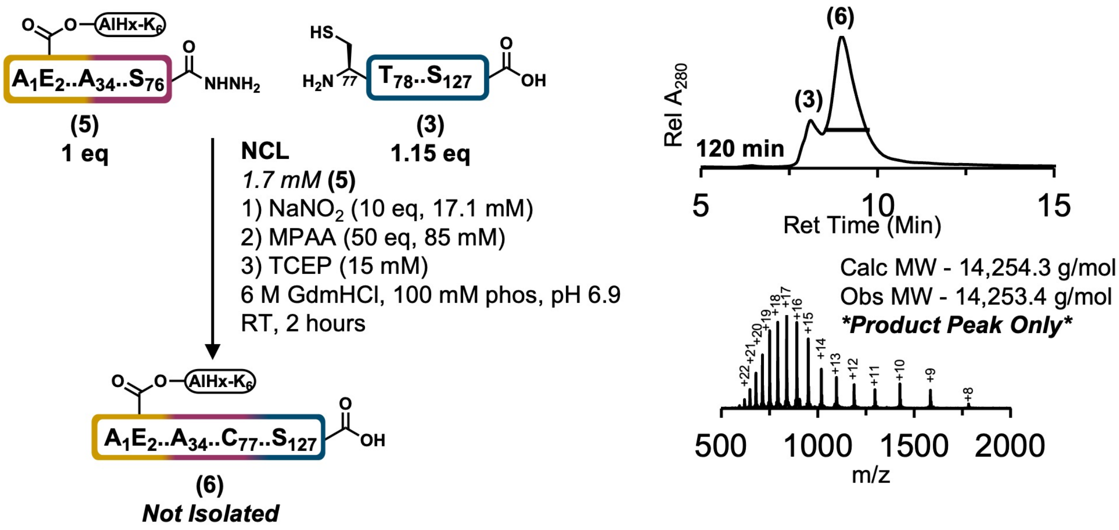

Figure S13. NCL of L-SA1-2(AlHx-K$_6$) **(5)** and L-SA3 **(3)**. Due to significant tailing and coelution of the peptides, this reaction was only monitored by LC/MS with LC/MS method C. After 120 min, no active thioester was detectable by MS. The MS shown is taken only from the TIC peak corresponding to the ligated product (shown in upper panel with black bar) to identify the ligated L-SA1-3(AlHx-K$_6$)_C77 **(6)** mass. This product was not purified and carried to the following desulfurization. See S8 for more detail.

(6) - AE(AlHxK$_6$)AGITGTWYNQLGSTFIVTAGADGALTGTYESAVGNAESRYVLTGRYDSAPA TDGSGTALGWTVAWKNNYRNAHSCTTWSGQYVGGAEARINTQWLLTSGTTEANAWKSTLV GHDTFTKVKPSAAS--OH

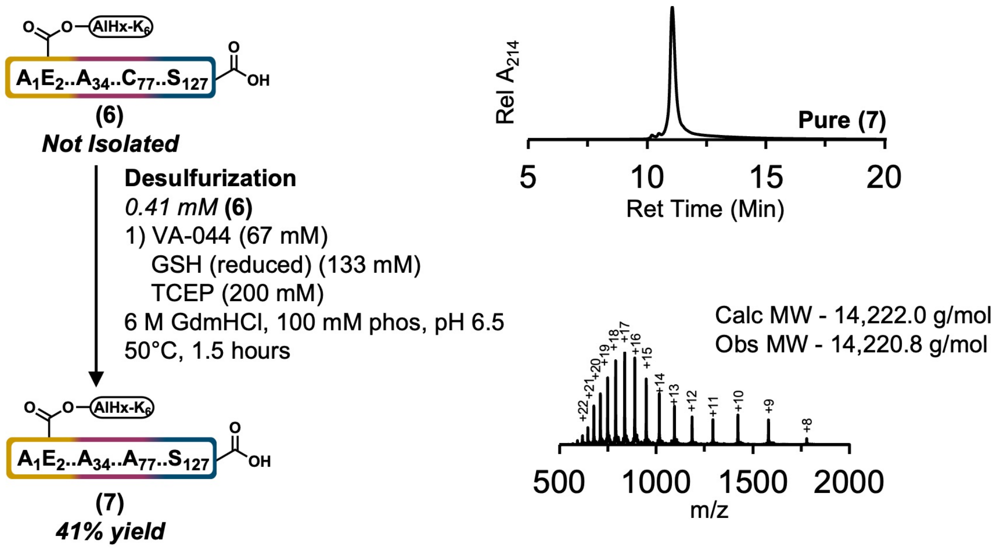

Figure S14. Desulfurization of L-SA1-3(AlHx-K$_6$)_C77 **(6)**. This reaction was monitored LC/MS with LC/MS method C. After 1.5 hours, no Cys remained, and the ligated product was purified. Pure L-SA1-3(AlHx-K$_6$) **(7)** RP-HPLC and MS traces are shown. See S8 for more detail.

(7) - A<u>E(AlHx-K$_6$)</u>AGITGTWYNQLGSTFIVTAGADGALTGTYESAVGNAESRYVLTGRYDSAPA TDGSGTALGWTVAWKNNYRNAHSATTWSGQYVGGAEARINTQWLLTSGTTEANAWKSTLVG HDTFTKVKPSAAS--OH

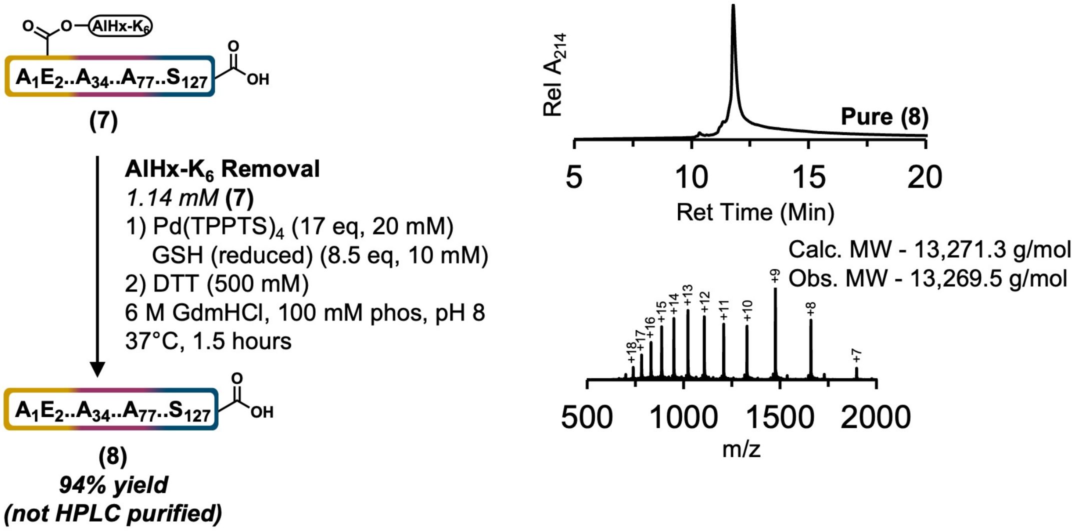

Figure S15. Removal of AlHx-K$_6$ from L-SA1-3(AlHx-K$_6$) **(7)**. This reaction was monitored LC/MS with LC/MS method C. After 1.5 hours, the reaction was quenched with DTT and subjected to dialysis to remove Pd and the AlHx-K$_6$ linker. L-SA1-3 **(8)** was not purified by RP-HPLC, and instead folded and characterized by CD, SEC, and ITC as described in S3, S11, and S12. RP-HPLC and MS traces of the pre-folded **(8)** after the final dialysis step are shown. See S8 for more detail.

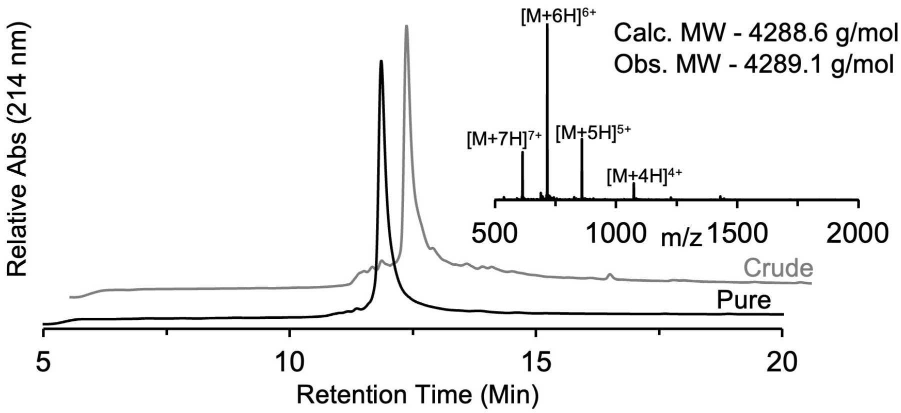

Figure S16. Characterization of D-SA1(AlHx-k_6) **(1*)**. This peptide was purified by purification method A with an isolated yield of 18.5% and analyzed by RP-HPLC with analytical method A. MS of the pure peptide is reported. See S9 for more detail.

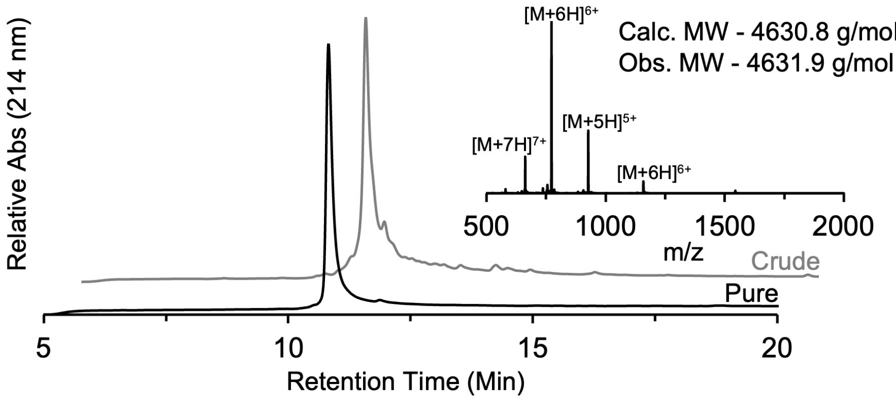

Figure S17. Characterization of D-SA2 **(2*)**. This peptide was purified by purification method B with an isolated yield of 19.1% and analyzed by RP-HPLC with analytical method A. MS of the pure peptide is reported. See S9 for more detail.

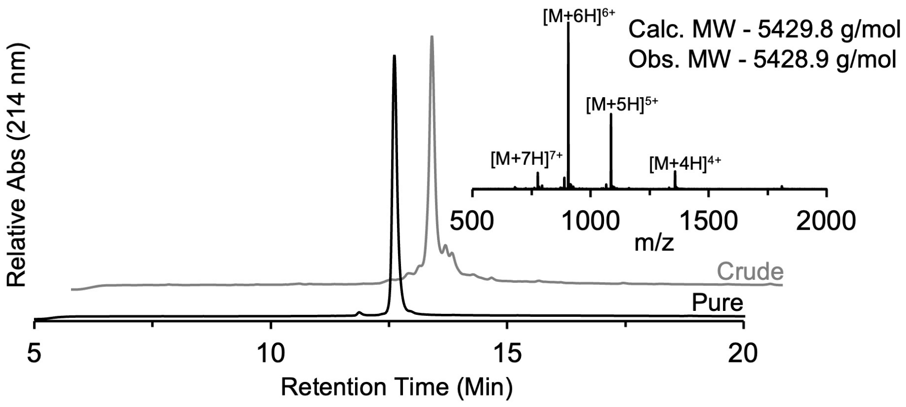

Figure S18. Characterization of D-SA3 **(3*)**. This peptide was purified by purification method C with an isolated yield of 19% and analyzed by RP-HPLC with analytical method A. MS of the pure peptide is reported. See S9 for more detail.

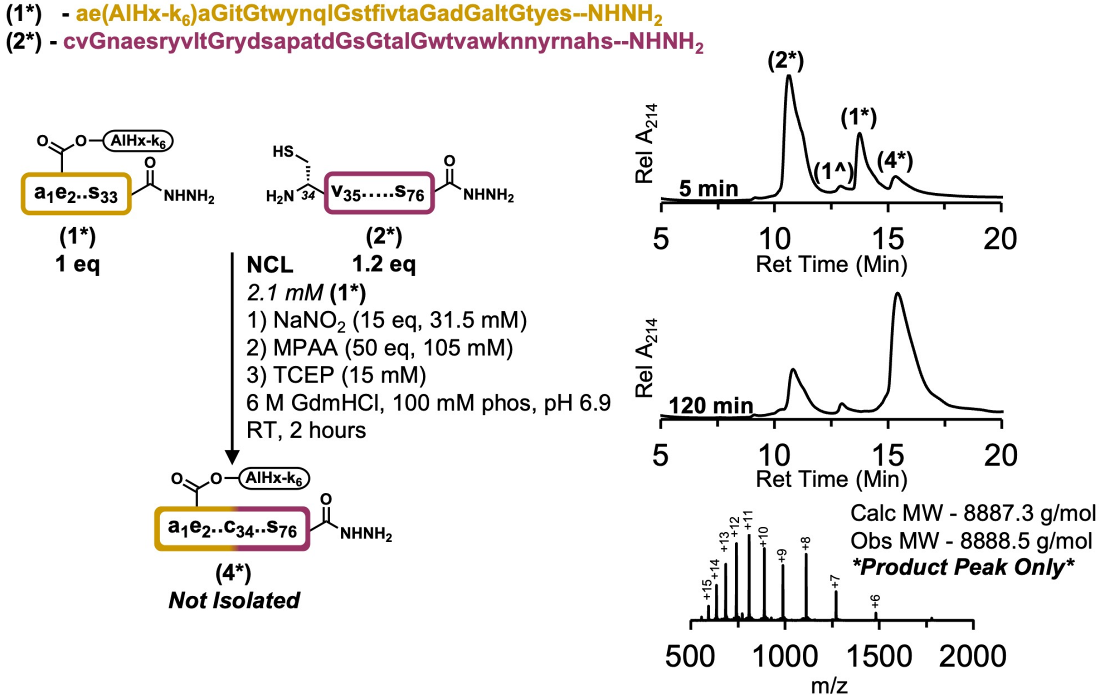

Figure S19. NCL of D-SA1(AlHx-k$_6$) **(1*)** and D-SA2 **(2*)**. This reaction was monitored by RP-HPLC with analytical method D. During the initial timepoint both **(1*)** thioester and hydrolyzed **(1^)** can be observed. After 120 min, no active MPAA thioester was present, and the reaction was stopped. The MS shown is taken only from the TIC peak corresponding to the ligated product D-SA1-2(AlHx-k$_6$)_c34 **(4*)** to identify the ligated mass. This product was not purified and carried to the following desulfurization. See S10 for more detail.

(4*) - ae(AlHx-k_6)aGitGtwynqlGstfivtaGadGaltGtyescvGnaesryvltGrydsapatdGsGtalGwtvawknn yrnahs--$NHNH_2$

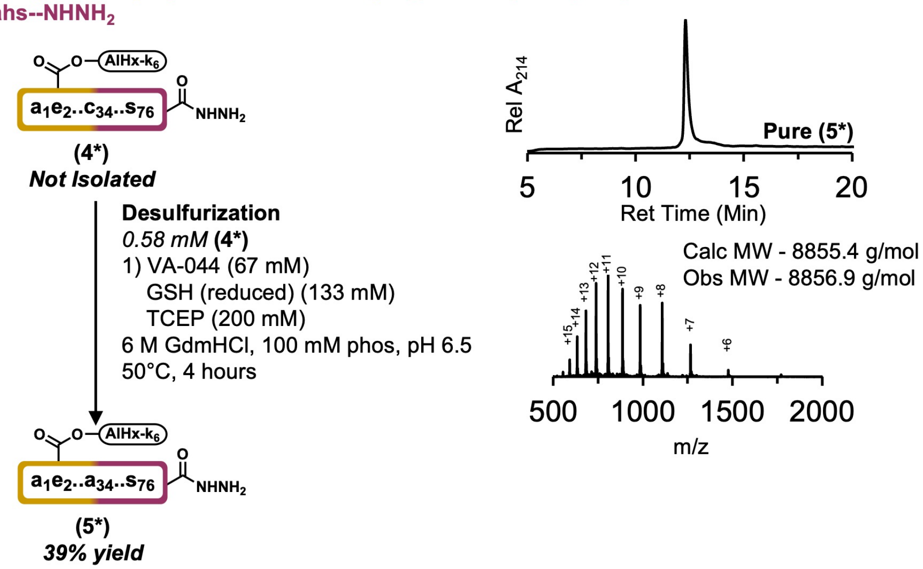

Figure S20. Desulfurization of D-SA1-2(AlHx-k_6)_c34 **(4*)**. This reaction was monitored LC/MS with LC/MS method B. After 4 hours, no additional desulfurization was observed, and the product D-SA1-2(AlHx-k_6) **(5*)** was purified. Pure **(5*)** RP-HPLC and MS traces are shown. See S10 for more detail.

(5*) - ae(AlHx-k_6)aGitGtwynqlGstfivtaGadGaltGtyesavGnaesryvltGrydsapatdGsGtalGwtvawknn yrnahs--$NHNH_2$
(3*) - cttwsGqyvGGaearintqwlltsGtteanawkstlvGhdtftkvkpsaas--OH

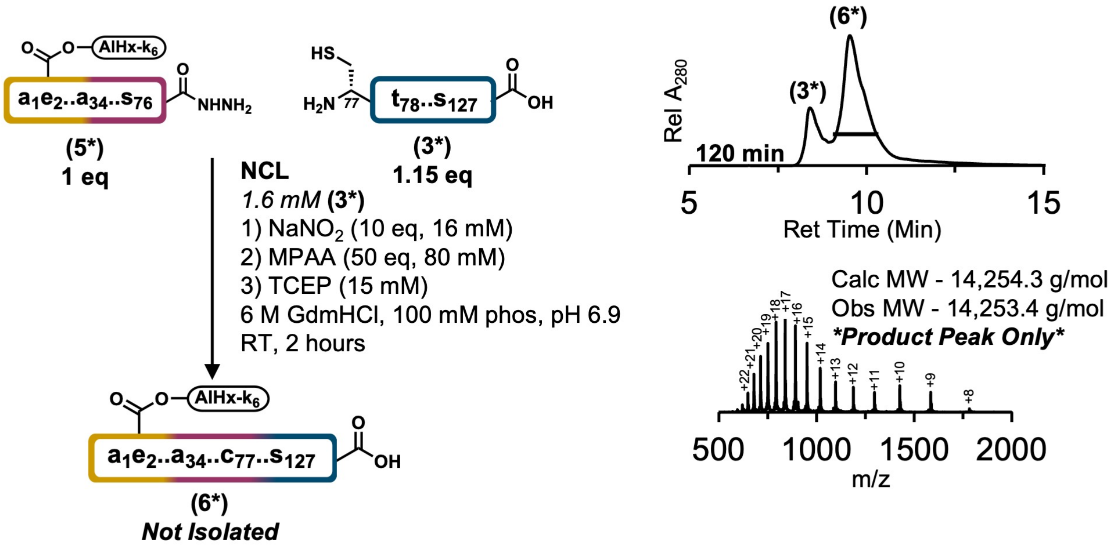

Figure S21. NCL of D-SA1-2(AlHx-k_6) **(5*)** and D-SA3 **(3*)**. Like the L-version, significant tailing and coelution of the peptides only allowed this reaction to be monitored by LC/MS with LC/MS method C. After 120 min, no active thioester was detectable by MS. The MS shown is taken only from the TIC peak corresponding to the ligated product (shown in upper panel with black bar) to identify the ligated mass D-SA1-3(AlHx-k_6)_c77 **(6*)**. This product was not purified and carried to the following desulfurization. See S10 for more detail.

(6*) - ae(AlHx-k$_6$)aGitGtwynqlGstfivtaGadGaltGtyesavGnaesryvltGrydsapatdGsGtalGwtvawknn yrnahs<u>c</u>ttwsGqyvGGaearintqwlltsGtteanawkstlvGhdtftkvkpsaas--OH

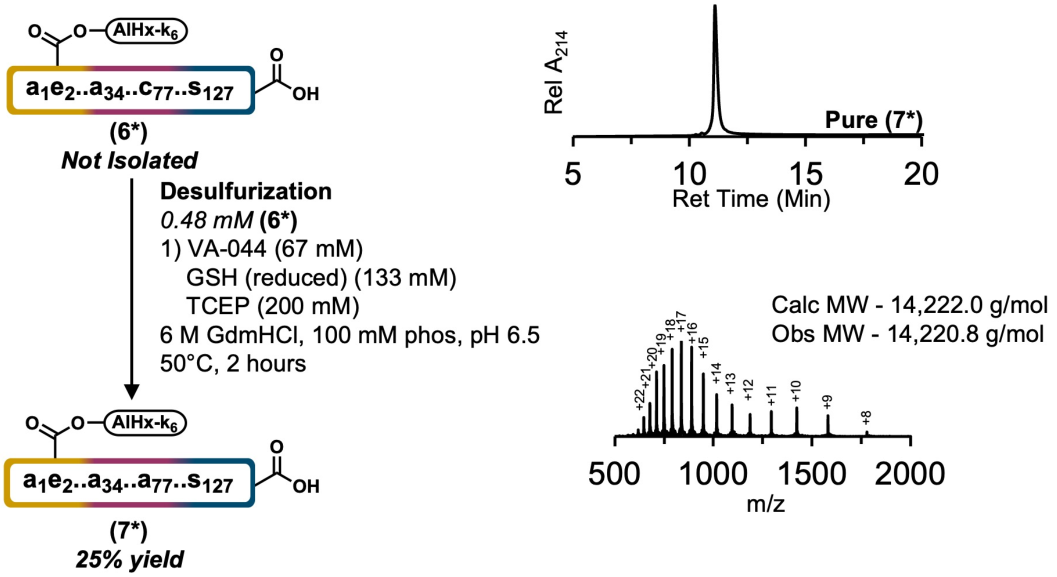

Figure S22. Desulfurization of D-SA1-3(AlHx-k$_6$)_c77 **(6*)**. This reaction was monitored LC/MS with LC/MS method C. After 1.5 hours, no Cys remained, and the ligated product was purified. Pure D-SA1-3(AlHx-k$_6$) **(7*)** RP-HPLC and MS traces are shown. See S10 for more detail.

(7*) - ae(AlHx-k6)aGitGtwynqlGstfivtaGadGaltGtyesavGnaesryvltGrydsapatdGsGtalGwtvawknn yrnahsattwsGqyvGGaearintqwlltsGtteanawkstlvGhdtftkvkpsaas--OH

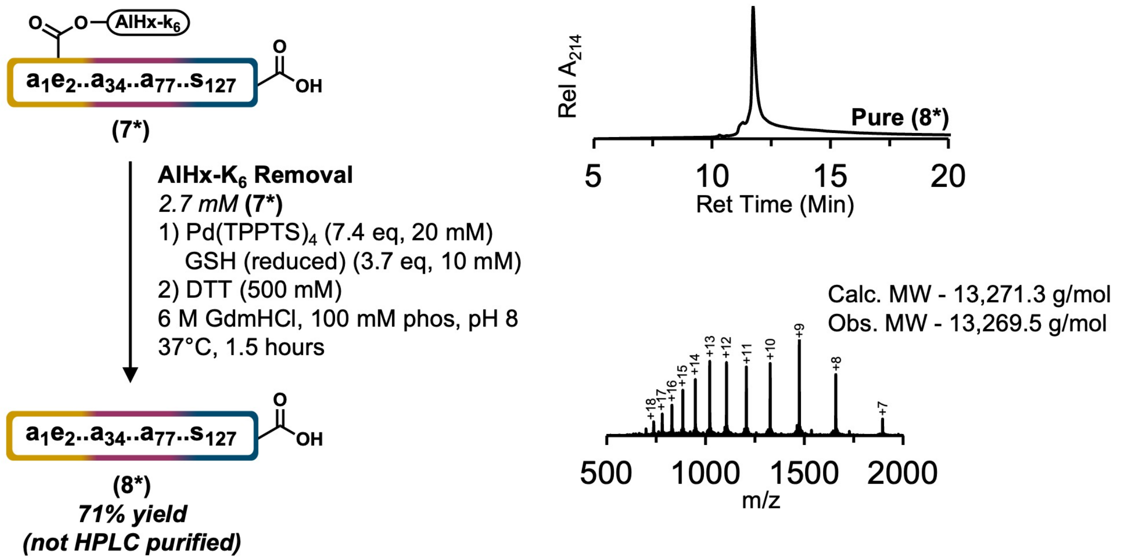

Figure S23. Removal of AlHx-k_6 from D-SA1-3(AlHx-k_6) **(7*)**. This reaction was monitored LC/MS with LC/MS method C. After 1.5 hours, the reaction was quenched with DTT and subjected to dialysis to remove Pd and the AlHx-k_6 linker. D-SA1-3 **(8*)** was not purified by RP-HPLC, and instead folded and characterized by CD, SEC, and ITC as described in S3, S11, and S12. RP-HPLC and MS traces of the pre-folded **(8*)** after the final dialysis step are shown. See S10 for more detail.

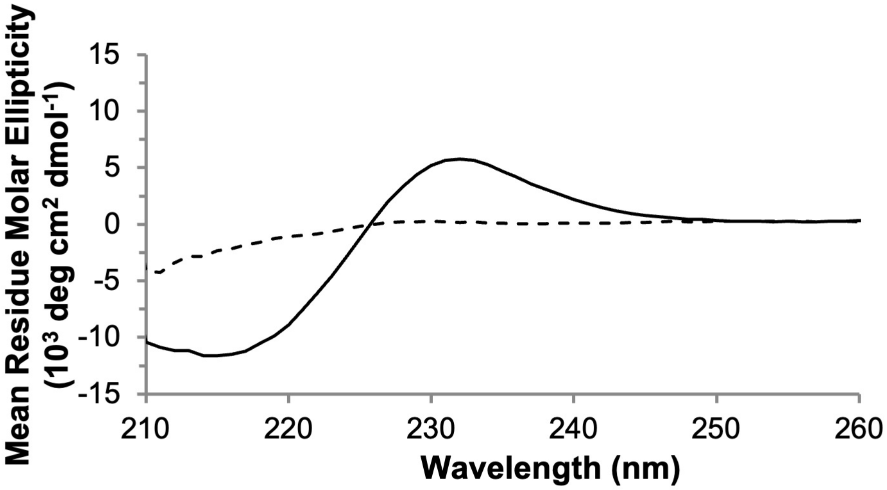

Figure S24. CD spectra of unfolded and refolded recombinant SA. The unfolded (dotted) line shows no structure and was then refolded (black line) using our HABA procedure.

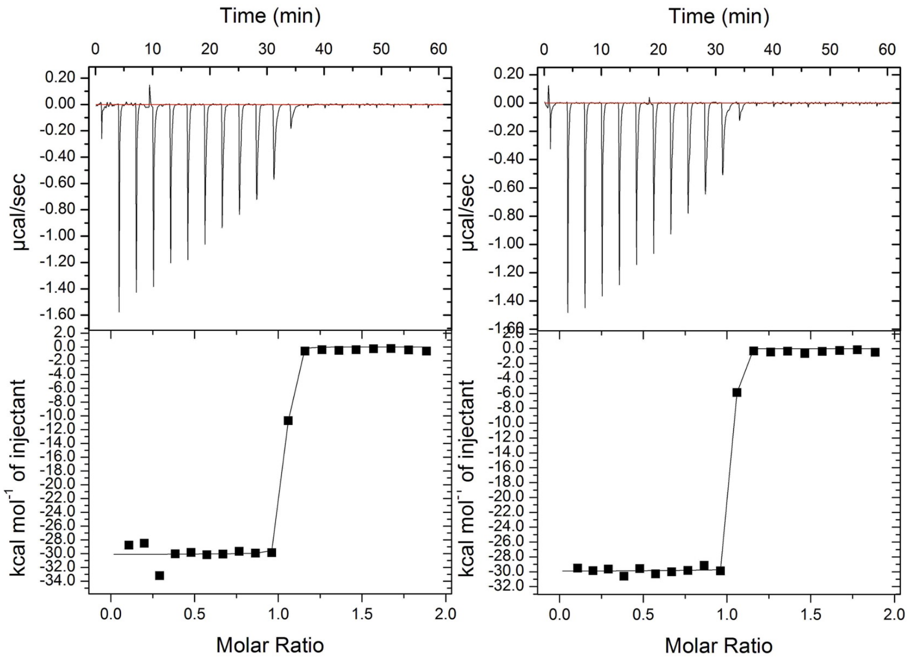

Figure S25. Duplicate ITC data for recombinant SA with (+)-biotin.

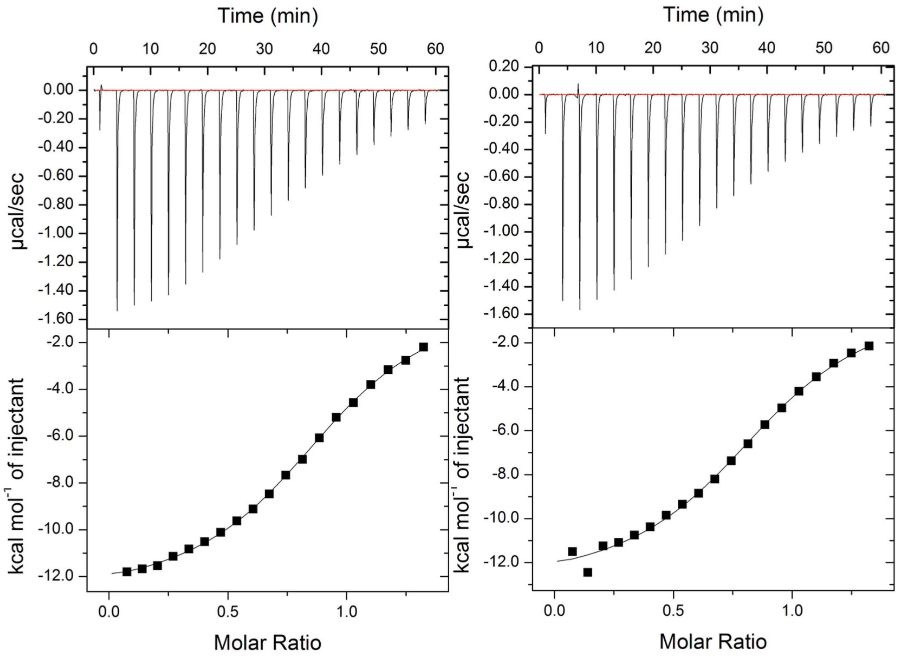

Figure S26. Duplicate ITC data for recombinant SA with (-)-biotin.

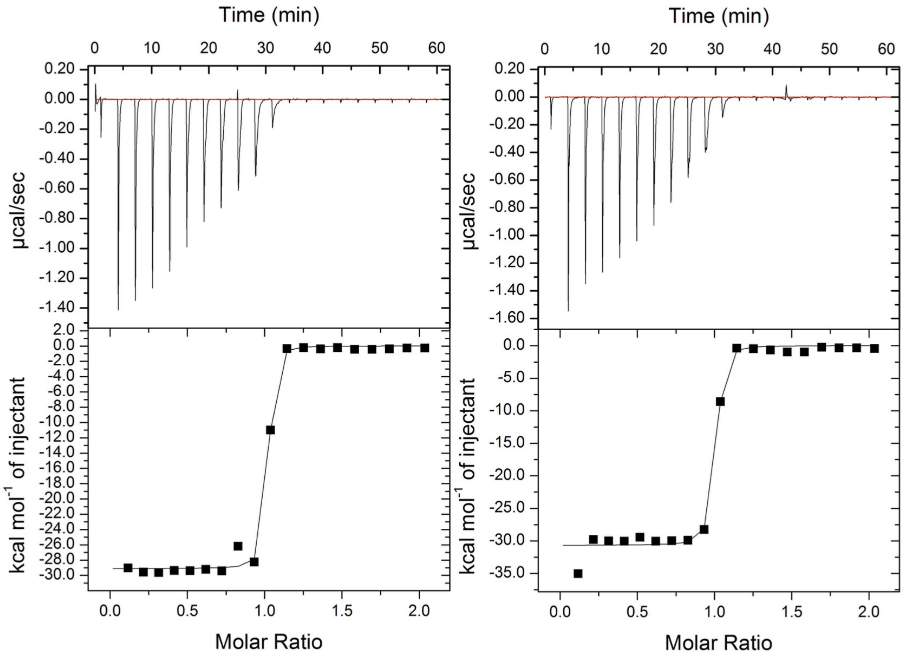

Figure S27. Duplicate ITC data for L-SA with (+)-biotin.

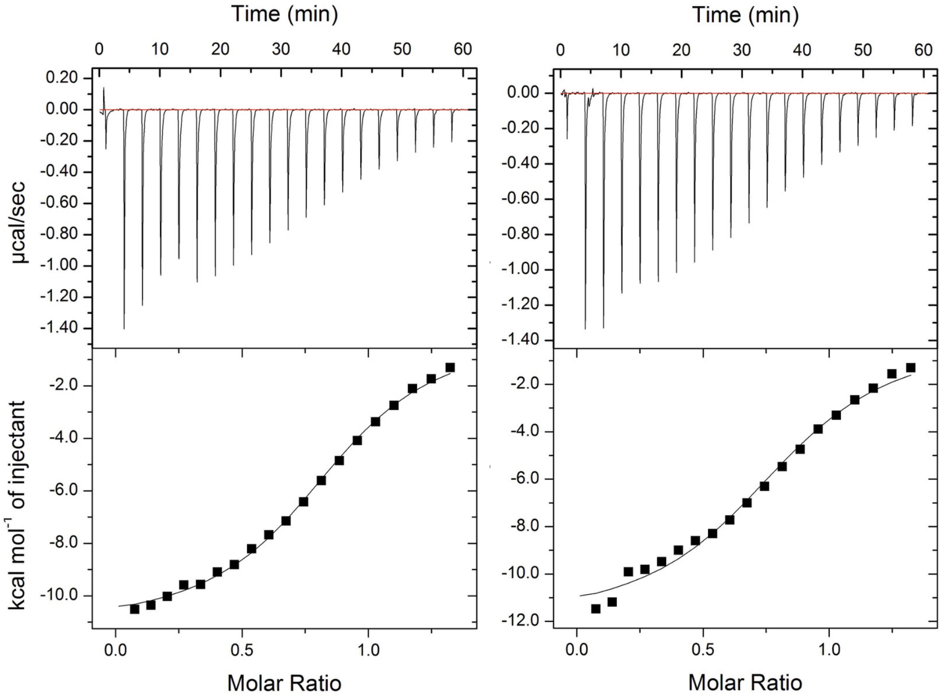

Figure S28. Duplicate ITC data for L-SA with (-)-biotin.

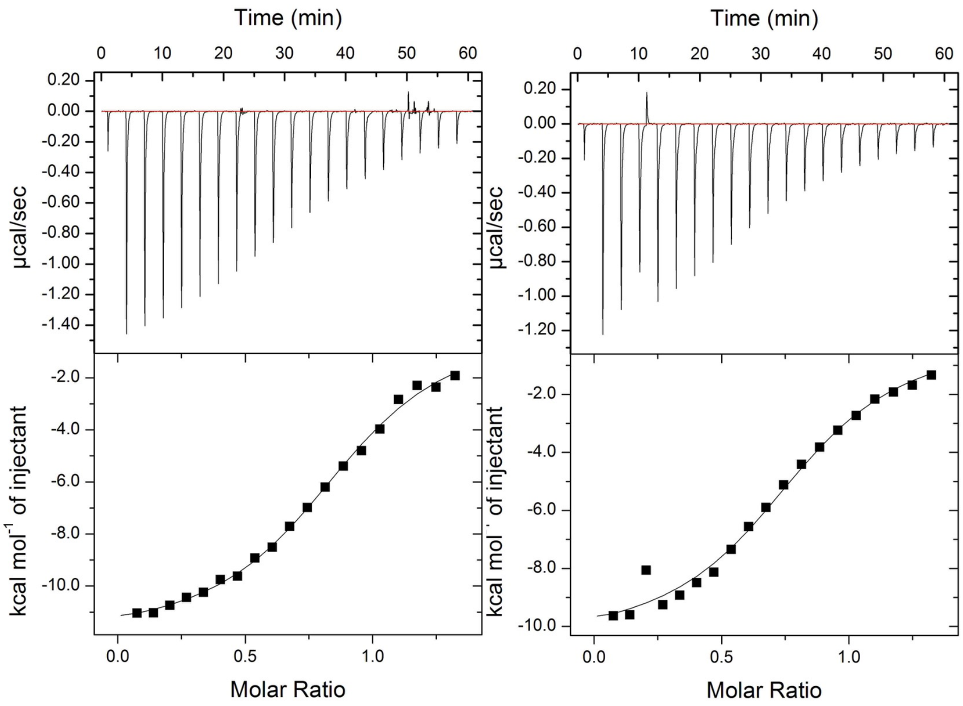

Figure S29. Duplicate ITC data for D-SA with (+)-biotin.

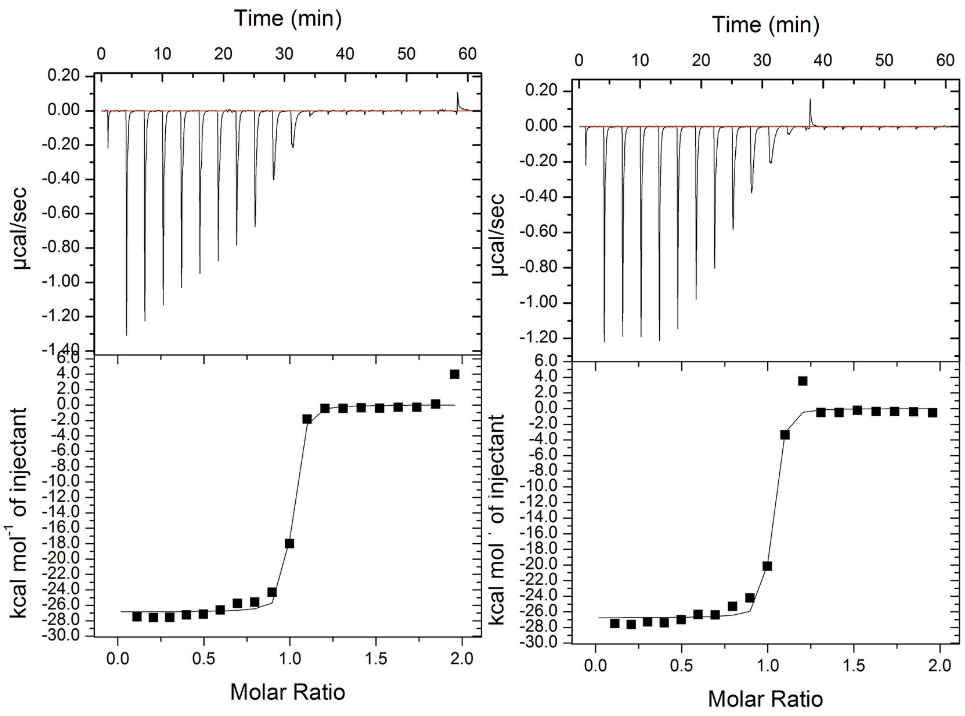

Figure S30. Duplicate ITC data for D-SA with (-)-biotin.

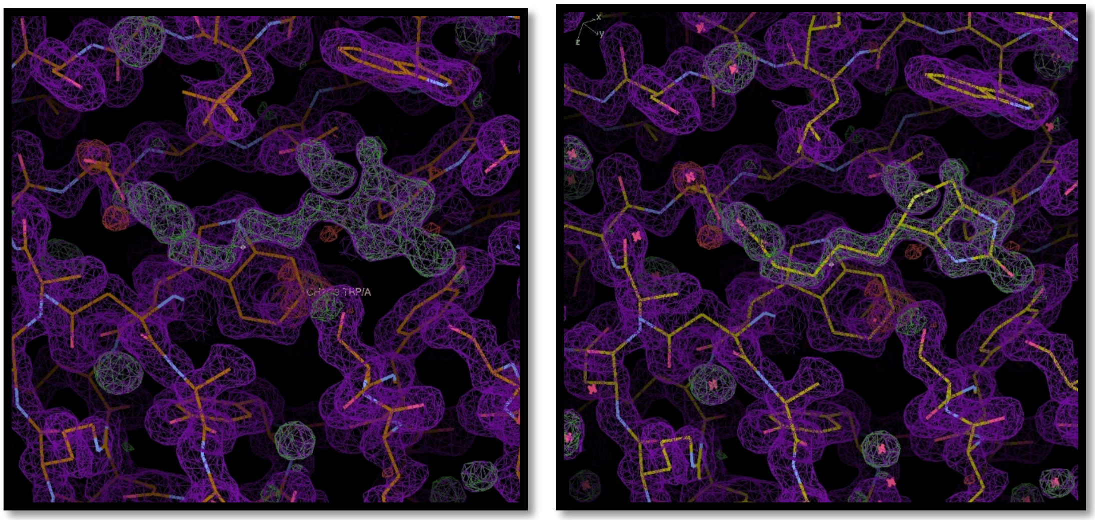

Figure S31. Unbiased electron density for (-)-biotin (left). (-)-biotin fit into electron density before refining (right).

Folding Method	Rec SA	L-SA	D-SA
Dilution-based	22% (n=2)	X	X
HABA (7.5 eq)	55% (n=2)	X	X
HABA (75 eq)	59% (n=2)	44% (n=1)	34% (n=1)
HABA (75 eq) (2mL)	X	53% (n=1)	35% (n=1)

Table S1. Folding Table for Recombinant, L-, and D-SA. Reported yields are based on A_{280} measurements of the final folded protein relative to the starting material. Trial numbers are shown in paretheses.

**Crystal Name – delete for publication**	**SABD04**	**SABL05**
**Ligand**	**D-Biotin (+)-biotin (BTN)**	**L-Biotin (-)-biotin (QTN)**
Source/Wavelength/Date of data collection	SSRL 9-2 / 0.96000 / 10/29/2022	SSRL 9-2 / 0.96000 / 10/29/2022
Detector	Pilatus 6M	Pilatus 6M
Program	XDS, AIMLESS	XDS, AIMLESS
Space Group (unit cell dimensions)	I222 (46.79, 94.60, 105.20)	I222 (46.69, 94.41, 105.14)
Resolution (Å)	41.94 – 0.95	41.85 – 0.94
Resolution (Å) (high-resolution shell)	(0.97 – 0.95)	(0.96 – 0.94)
# Reflections measured	4926376 (102593)	4899462 (106635)
# Unique reflections	140605 (5670)	139644 (5222)
Redundancy (high-resolution shell)	35 (18)	35 (20)
Completeness (%)(high-resolution shell)	96.8 (79.3)	93.7 (71.6)
<I/σI> (high-resolution shell)	31.7 (0.8)	31.4 (0.8)
<CC1/2>	1.000 (0.354)	1.000 (0.449)
Rpim (high-resolution shell)	0.010 (0.718)	0.009 (0.798)
Mosaicity (°)	0.11	0.15
**Refinement**		
**Crystal Name – refinement cycle**	**SABD04_refmac_7**	**SABL05_refine_30**
**Abbreviated name**	**DRMR7**	**L05R30**
Program	Refmac5	Refmac5
Resolution (Å)	41.89 – 0.95	41.89– 0.94
Resolution (Å) – (high-resolution shell)	(0.977 – 0.953)	(0.954 – 0.944)
# Reflections	136831 (8351)	136221 (7710)
# Reflections (%) in Rfree set	2793 (2.0%)	2769 (2.0%)
Rcryst[b]	0.135 (0.691)	0.131 (0.541)
Rfree[c]	0.148 (0.783)	0.149 (0.510)
RMSD: bonds (Å) / angles (°)	0.025 / 2.308	0.028 / 2.377
<B> ($Å^2$): all atoms / # atoms	13.1 / 3957	17.4 / 3943
<B> ($Å^2$): Biotin molecules / #Biotin	6.6 / 2	9.4 / 2 in 2 conf
<B> ($Å^2$): water molecules / #water	28.9 / 314	30.1 / 314
<B> ($Å^2$): other solvent molecules / #Solvent	29.9 / 1	33.8 / 1
ϕ/ψ most favored (%) / additionally allowed (%)	98.3/1.7	98.3/1.7
Coordinate error ($Å^2$)	0.010	0.011

Table S2. Crystallography table for recombinant SA complexed with (+)- and (-)-biotin.

(1) Giesler, R. J.; Spaltenstein, P.; Jacobsen, M. T.; Xu, W.; Maqueda, M.; Kay, M. S. A glutamic acid-based traceless linker to address challenging chemical protein syntheses. *Org Biomol Chem* **2021**, *19* (40), 8821-8829. DOI: 10.1039/d1ob01611c.
(2) Jacobsen, M. T.; Spaltenstein, P.; Giesler, R. J.; Chou, D. H.; Kay, M. S. Improved Handling of Peptide Segments Using Side Chain-Based "Helping Hand" Solubilizing Tools. *Methods Mol Biol* **2022**, *2530*, 81-107. DOI: 10.1007/978-1-0716-2489-0_7 From NLM Medline.
(3) Huang, Y. C.; Chen, C. C.; Li, S. J.; Gao, S.; Shi, J.; Li, Y. M. Facile synthesis of C-terminal peptide hydrazide and thioester of NY-ESO-1 (A39-A68) from an Fmoc-hydrazine 2-chlorotrityl chloride resin. *Tetrahedron* **2014**, *70* (18), 2951-2955.
(4) Spaltenstein, P.; Giesler, R. J.; Scherer, S. R.; Erickson, P. W.; Kay, M. S. Selective Activation of Peptide-Thioester Precursors for Templated Native Chemical Ligations. *Angewandte Chemie* **2024**, e202413644.
(5) Suganuma, M.; Kubo, T.; Ishiki, K.; Tanaka, K.; Suto, K.; Ejima, D.; Toyota, M.; Tsumoto, K.; Sato, T.; Nishikawa, Y. Mirror-image streptavidin with specific binding to L-biotin, the unnatural enantiomer. *Sci Rep* **2022**, *12* (1), 9568. DOI: 10.1038/s41598-022-13763-4.
(6) Soltis, S. M.; Cohen, A. E.; Deacon, A.; Eriksson, T.; Gonzalez, A.; McPhillips, S.; Chui, H.; Dunten, P.; Hollenbeck, M.; Mathews, I.; et al. New paradigm for macromolecular crystallography experiments at SSRL: automated crystal screening and remote data collection. *Acta Crystallogr D Biol Crystallogr* **2008**, *64* (Pt 12), 1210-1221. DOI: 10.1107/S0907444908030564 From NLM Medline.
(7) McPhillips, T. M.; McPhillips, S. E.; Chiu, H. J.; Cohen, A. E.; Deacon, A. M.; Ellis, P. J.; Garman, E.; Gonzalez, A.; Sauter, N. K.; Phizackerley, R. P.; et al. Blu-Ice and the Distributed Control System: software for data acquisition and instrument control at macromolecular crystallography beamlines. *J Synchrotron Radiat* **2002**, *9* (Pt 6), 401-406. DOI: 10.1107/s0909049502015170 From NLM Medline.
(8) Russi, S.; Song, J.; McPhillips, S. E.; Cohen, A. E. The Stanford Automated Mounter: pushing the limits of sample exchange at the SSRL macromolecular crystallography beamlines. *J Appl Crystallogr* **2016**, *49* (Pt 2), 622-626. DOI: 10.1107/S1600576716000649 From NLM PubMed-not-MEDLINE.
(9) Cohen, A. E.; Ellis, P. J.; Miller, M. D.; Deacon, A. M.; Phizackerley, R. P. An automated system to mount cryo-cooled protein crystals on a synchrotron beam line, using compact sample cassettes and a small-scale robot. *J Appl Crystallogr* **2002**, *35* (6), 720-726. DOI: 10.1107/s0021889802016709 From NLM PubMed-not-MEDLINE.
(10) Kabsch, W. Xds. *Acta Crystallogr D Biol Crystallogr* **2010**, *66* (Pt 2), 125-132. DOI: 10.1107/S0907444909047337 From NLM Medline.
(11) Evans, P. R. An introduction to data reduction: space-group determination, scaling and intensity statistics. *Acta Crystallogr D Biol Crystallogr* **2011**, *67* (Pt 4), 282-292. DOI: 10.1107/S090744491003982X From NLM Medline.
(12) Liebschner, D.; Afonine, P. V.; Baker, M. L.; Bunkoczi, G.; Chen, V. B.; Croll, T. I.; Hintze, B.; Hung, L. W.; Jain, S.; McCoy, A. J.; et al. Macromolecular structure determination using X-rays, neutrons and electrons: recent developments in Phenix. *Acta Crystallogr D Struct Biol* **2019**, *75* (Pt 10), 861-877. DOI: 10.1107/S2059798319011471 From NLM Medline.
(13) Emsley, P.; Cowtan, K. Coot: model-building tools for molecular graphics. *Acta Crystallogr D Biol Crystallogr* **2004**, *60* (Pt 12 Pt 1), 2126-2132. DOI: 10.1107/S0907444904019158 From NLM Medline.
(14) Afonine, P. V.; Grosse-Kunstleve, R. W.; Echols, N.; Headd, J. J.; Moriarty, N. W.; Mustyakimov, M.; Terwilliger, T. C.; Urzhumtsev, A.; Zwart, P. H.; Adams, P. D. Towards automated crystallographic structure refinement with phenix.refine. *Acta Crystallogr D Biol Crystallogr* **2012**, *68* (Pt 4), 352-367. DOI: 10.1107/S0907444912001308.

(15) Murshudov, G. N.; Vagin, A. A.; Dodson, E. J. Refinement of macromolecular structures by the maximum-likelihood method. *Acta Crystallogr D Biol Crystallogr* **1997**, *53* (Pt 3), 240-255. DOI: 10.1107/S0907444996012255.
(16) Kabsch, W. A solution for the best rotation to relate two sets of vectors. *Acta Crystallographica Section A: Crystal Physics, Diffraction, Theoretical and General Crystallography* **1976**, *32* (5), 922-923.
(17) Potterton, E.; Briggs, P.; Turkenburg, M.; Dodson, E. A graphical user interface to the CCP4 program suite. *Acta Crystallogr D Biol Crystallogr* **2003**, *59* (Pt 7), 1131-1137. DOI: 10.1107/s0907444903008126 From NLM Medline.
